# Drug-Drug interactions prediction calculations between cardiovascular drugs and antidepressants for discovering the potential co-medication risks

**DOI:** 10.1371/journal.pone.0316021

**Published:** 2025-01-13

**Authors:** Tie Hua Zhou, Tian Yu Jin, Xi Wei Wang, Ling Wang

**Affiliations:** Department of Computer Science and Technology, School of Computer Science, Northeast Electric Power University, Jilin, China; King Abdulaziz University, SAUDI ARABIA

## Abstract

Predicting Drug-Drug Interactions (DDIs) enables cost reduction and time savings in the drug discovery process, while effectively screening and optimizing drugs. The intensification of societal aging and the increase in life stress have led to a growing number of patients suffering from both heart disease and depression. These patients often need to use cardiovascular drugs and antidepressants for polypharmacy, but potential DDIs may compromise treatment effectiveness and patient safety. To predict interactions between drugs used to treat these two diseases, we propose a method named Multi-Drug Features Learning with Drug Relation Regularization (MDFLDRR). First, we map feature vectors representing drugs in different feature spaces to the same. Second, we propose drug relation regularization to determine drug pair relationships in the interaction space. Experimental results demonstrate that MDFLDRR can be effectively applied to two DDI prediction goals: predicting unobserved interactions among drugs within the drug network and predicting interactions between drugs inside and outside the network. Publicly available evidence confirms that MDFLDRR can accurately identify DDIs between cardiovascular drugs and antidepressants. Lastly, by utilizing drug structure calculations, we ascertained the severity of newly discovered DDIs to mine the potential co-medication risks and aid in the smart management of pharmaceuticals.

## Introduction

A drug is a chemical or natural substance employed for disease prevention, diagnosis, treatment and symptom relief. Drug-Drug Interaction (DDI) refers to the changes in PharmacoDynamics (PD) and PharmacoKinetics (PK) that occur when one drug is administered concurrently or at certain intervals with another drug or multiple other drugs [1, 2]. The rational use of DDIs can enhance drug efficacy, alleviate or avoid the toxic side effects of drugs; conversely, it could lead to an increase in toxic side effects or a decrease in drug efficacy, even worsening the condition or endangering life [3–5].

Prediction of DDIs is carried out through bioinformatics models and algorithms. These models and algorithms can simulate the dynamic behavior and interactions of drugs within biological entities. This predictive method allows for a more efficient understanding of a drug’s potential effects, facilitates discovery of new drug action mechanisms, and can identify combinations of drugs that may produce synergistic effects. This significantly accelerates the drug discovery process. By predicting DDIs, researchers can screen out potential drug combinations that may cause adverse reactions at early stages, thereby enhancing the safety and efficiency of drug development, serving the purpose of reducing the time required for drug approval. In addition, prediction of DDIs has a close relationship with drug repositioning and drug repurposing. Drug repositioning refers to discovering new uses for existing drugs, whereas drug repurposing involves using existing drugs to treat new diseases. Through predicting DDIs, not only can we identify new drug combinations, but we can also understand how different drugs interact. This can lead to discovery of a certain drug’s potential utility in treating new diseases. For instance, a drug initially used for treating heart disease may demonstrate effects in treating neurodegenerative diseases when it interacts with other drugs. Discovering such mechanisms can accelerate drug repositioning and repurposing, providing more possibilities for medical research and treatment.

Globally, heart disease and depression are two prevalent conditions [6–8]. Heart disease primarily refers to diseases related to the heart, such as hypertension, arrhythmia, coronary artery disease and valvular heart disease [9–11]. Depression, on the other hand, is a psychological disease, manifesting symptoms such as tremendous pressure, loss of interest in life, persistent feelings of sadness, helplessness and changes in sleep and appetite [12–14]. Although these two diseases are of different types, in many cases, they appear together in the same patient, causing greater health stress. Medication is a very important part of the treatment for these two diseases. For heart disease, drugs such as antihypertensives, anticoagulants, antiplatelets, and anti-arrhythmics are commonly used [15]. Common drugs for depression include Tricyclic Antidepressants (TCA), Monoamine Oxidase Inhibitors (MAOI) and Selective Serotonin Reuptake Inhibitors (SSRI) [16]. However, when heart disease and depression coexist, the appropriateness of polypharmacy will directly lead to different outcomes. This is because the combined use of cardiovascular drugs and antidepressants may result in DDIs, leading to Adverse Drug Reactions (ADR) [17]. For instance, certain antidepressants may affect the electrophysiological properties of the heart, increasing the risk of arrhythmia, which is a huge risk for those already suffering from heart disease [18].

The popularity of polypharmacy is growing with the influx of new drugs on the market, and how to efficiently and accurately determine unknown DDIs to reduce the frequency of ADRs has become an urgent problem to solve [19–21]. However, traditional wet-lab methods require a lot of labor and the process takes a long time, and also have to consider animal welfare and ethical issues [3, 22]. Most of these methods involve evaluating standard doses for short-term use in order to determine whether they are effective or safe, easily overlooking DDIs that only emerge with long-term use of large doses of drugs. Therefore, only a limited number of DDIs can be detected by researchers during clinical trial phases, and more official reports on DDIs are typically released after the drug has been approved by FDA [23, 24]. In order to resolve these issues, scholars in computational biology and bioinformatics propose using advanced computer technologies to predict unobserved DDIs [25].

In this paper, we propose Multi-Drug Features Learning with Drug Relation Regularization (MDFLDRR) for predicting the interaction relationship between different drugs, especially cardiovascular drugs and antidepressants. The method can be roughly divided into two stages: In the first stage, we select the pathway, enzyme, target, and chemical substructure as the drug features needed for the experiment, and convert the feature space formed by drugs based on different drug features into a common interaction space. In the second stage, we propose a brand new method named Drug Relation Regularization (DRR) aimed at describing the relationship of drug pairs using the DDIs already present in the original data set. By mathematizing the problem, we not only define the objective function of MDFLDRR but also address optimization problems by further designing relevant schemes. Through a comprehensive multi-index analysis of our experimental results, we’ve found that the predictive advantage of MDFLDRR is extremely pronounced when compared to currently advanced comparison approaches. Our research and analysis of cardiovascular drugs and antidepressants led us to discover that MDFLDRR identified numerous new DDIs that could be corroborated by publicly available materials. Lastly, we tackled the issue of ascertaining the severity level of DDIs by generating molecular fingerprints using the Simplified Molecular Input Line Entry System (SMILES) and calculating the drug molecular structure similarity. Furthermore, these conclusions have been validated through different drug databases, ensuring their authenticity and effectiveness.

## Related work

Current computational approaches for detecting DDIs fall into three types: matrix factorization, network propagation and ensemble learning. Next, we will introduce the representative algorithms for each method in turn.

### Matrix factorization

Matrix factorization provides the mathematical foundation for constructing models in fields of computational biology and bioinformatics [26]. Matrix factorization methods are techniques for decomposing a matrix into several simpler matrices, used to extract latent features and reconstruct matrices to solve various problems, such as discovering unknown DDIs and dimension reduction. Vilar et al. [27] utilized Interaction Profile Fingerprints (IPF) to detect DDIs. In their study, they first constructed a drug interaction matrix *M*_1_, converted the collected DDIs into a binary matrix, and then generated a similarity matrix *M*_2_ of interaction configurations. Finally, they obtained matrix *M*_3_ by multiplying matrix *M*_1_ with matrix *M*_2_, from which they extracted predicted drug interactions, and associated the effects of biology attributed to both known DDIs and new DDIs. Shtar et al. [28] utilized Adjacency Matrix Factorization (AMF) to detect DDIs. This method generates a similarity matrix and decomposes the interaction matrix into two matrices as constraints. By multiplying these two matrices, new DDIs can be detected.

### Network propagation

Methods of this type predict DDIs by analyzing known DDI networks or drug molecular structure networks, using principles of information propagation and network topology [29]. Common network propagation methods fall into two categories: graph embedding and link prediction. Graph embedding is a technique that maps nodes and edges in a graph to a low-dimensional vector space, used to capture relationships and features between nodes for subsequent graph analysis and machine learning tasks. Marinka et al. [30] constructed an architecture for predicting DDIs using Graph Convolutional Networks (GCN). They combined Protein-Protein Interaction (PPI), Protein-Drug Interaction (PDI), and DDI to build a multi-model graph. By predicting multi-relation connections through this architecture, interactions and types of action between drugs can be determined. Ma et al. [31] integrated drug similarities using a multi-view graph autoencoder, and selected views through an attention mechanism to enhance the interpretability of the experiment. They modeled each drug with GCN, serving as a node embedded in multi-view node features and edges. Feng et al. [32] extracted structural drug features from the DDI network and predicted DDIs using a combination of GCN and Deep Neural Network (DNN). Link prediction is a method that predicts connections between nodes that do not yet exist by analyzing network topology. Park et al. [33] employed signal propagation and PPI comparisons to detect DDIs. They simulated signal propagation and captured the possibility of remote interference using a random walk and restart algorithm. By calculating the probability and overlap effects of proteins, they obtained a metric, DDIScore, to quantify the credibility of DDIs.

### Ensemble learning

Ensemble learning enhances the accuracy and robustness of DDI prediction by combining the results of multiple prediction models, leveraging the synergy and diversity between models. Zhang et al. [34] proposed FSMLKNN, a method aimed at reducing the computational burden in multi-label situations by selecting appropriate information dimensions is used to predict DDIs. Lin et al. [35] proposed a novel method, MDF-SA-DDI, which predicts DDIs events by integrating multi-source drug fusion, multi-source feature fusion, and the transformer self-attention mechanism. Deepika et al. [36] constructed a Semi-supervised classifier to detect DDIs. This method combines the advantages of Positive Unlabeled learning and Meta-learning. It can effectively utilize limited labeled data for reasonable DDI prediction, which can further enhance prediction performance.

## Motivation and overall workflow design

As introduced earlier, quite a number of researchers have been involved in developing individual machine learning methods for predicting DDIs. However, most methods directly choose the drug data set from the drug database for various diseases to predict DDIs, and do not delve into the interaction relationship between drugs treating two specific diseases. This also results in the proposed methods being unable to be directly applied to the corresponding disease fields. Meanwhile, in the topic of DDI prediction, the existing bioinformatics and computational biology methods have failed to further infer the severity levels of predicted DDIs, which has emerged as a new research focus within this subject. In addition, to enhance the authenticity and reasonability of DDI prediction, we have summarized three points regarding the shortcomings of most machine learning methods. First, many existing prediction methods only involve the use of a single drug feature, such as molecular fingerprinting, while there are very few methods that further enhance prediction accuracy by combining various different drug features. Second, most methods predict unknown interactions by using known ones, but the drawback of this method is that it is not suitable for predicting drugs with no known interactions. Third, the classifiers used in methods based on ensemble learning use known pairs of drugs that will not interact as negative samples. However, this method is highly susceptible to false negatives in data, thus compromising some accuracy.

**Fig 1:**
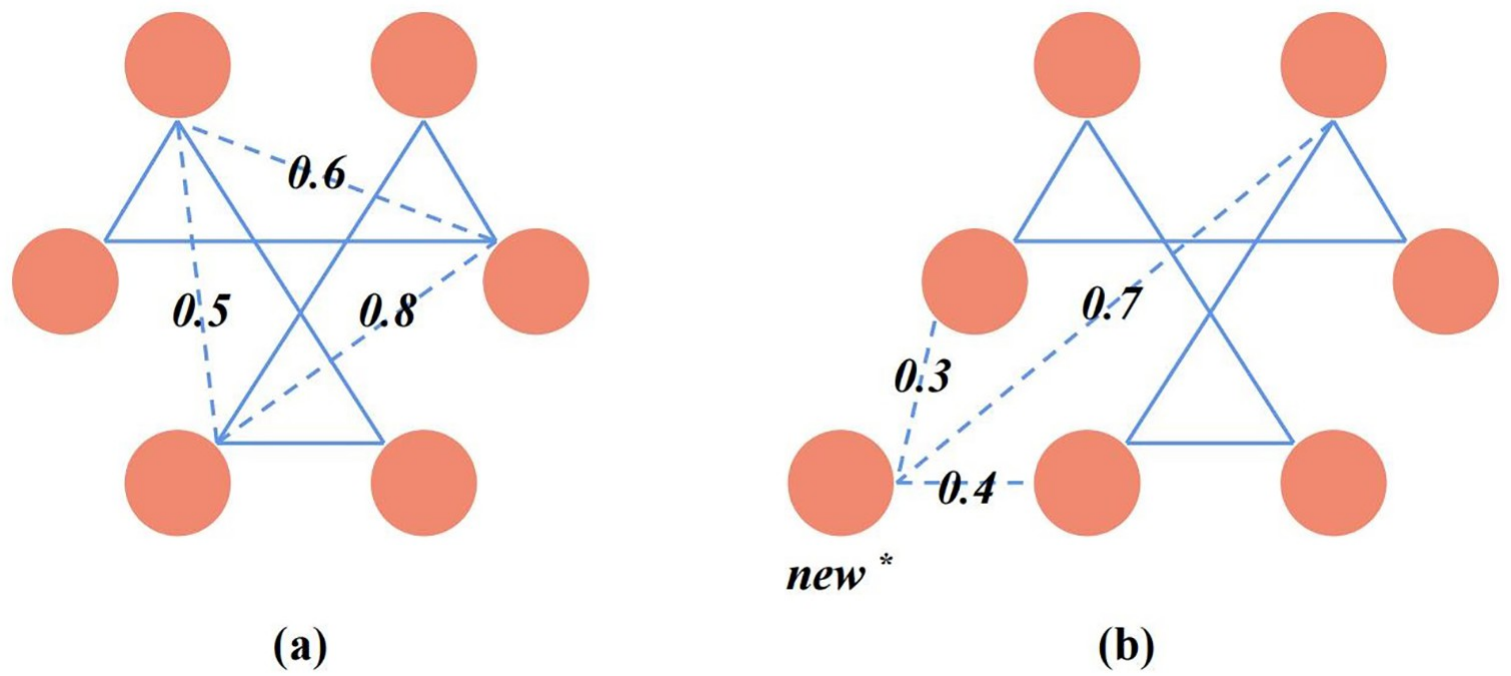


To facilitate the description of the workflow, we have defined the following two prediction goals and visually presented them in Fig 1.

**Goal 1.** *Predicting unobserved interactions among known drugs within the drug network*.
**Goal 2.** *Predicting unobserved interactions between known drugs inside the drug network and new drugs outside the drug network*.

The total number of drugs included in the data set is represented by *n*, and the known DDIs are represented by the interaction matrix *J*. If drug *i* interacts with drug *j*, we denote it as *J*_*ij*_ = 1, otherwise, it’s 0. By using *m* different drug features, *n* drugs are transformed into *m* × *n* feature vectors. The *n* feature vectors obtained based on the same drug feature form a feature matrix *H*. Therefore, the feature matrices formed by using *m* different drug features are represented as ${\left\{H^{i}\right\}}_{i=1}^{m}$.

### Overall workflow design

**Fig 2:**
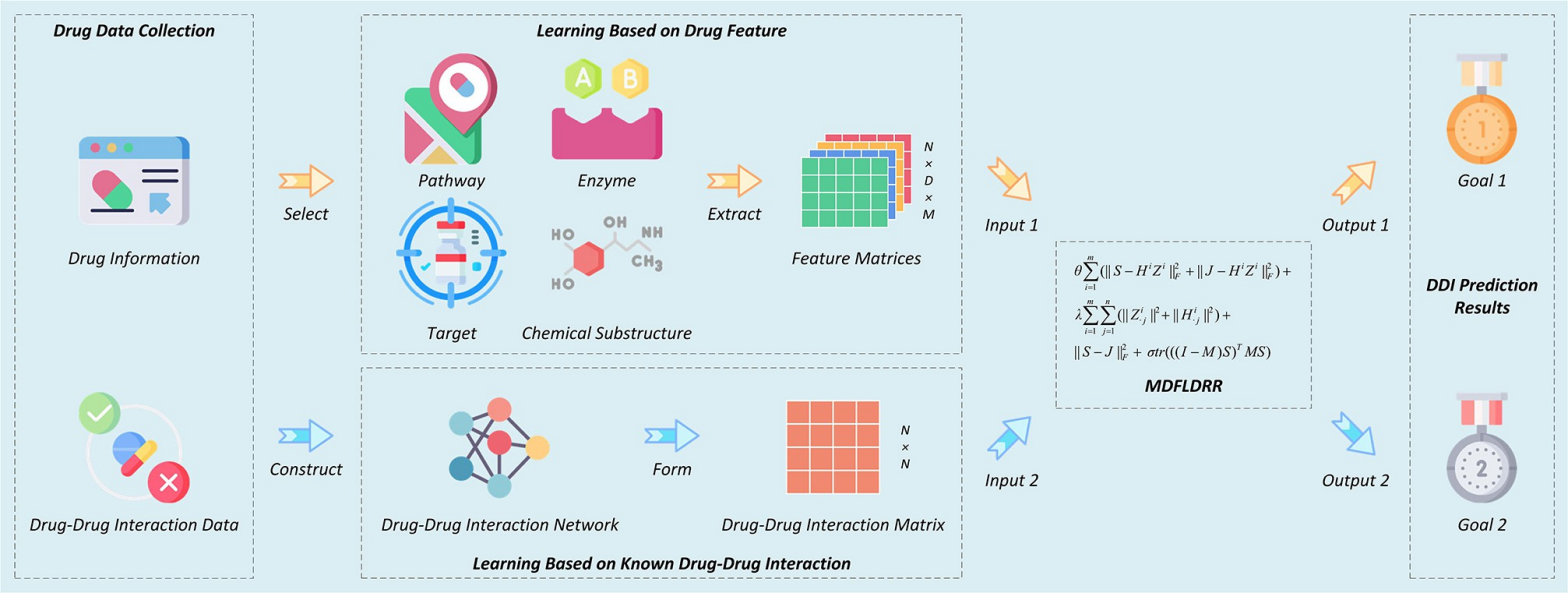


In this research, we propose Multi-Drug Features Learning with Drug Relation Regularization (MDFLDRR) for predicting DDIs, as shown in Fig 2. First, we map the *m* drug feature matrices ${\left\{H^{i}\right\}}_{i=1}^{m}$ to an interaction matrix *S*, and in combination with the proposed Drug Relation Regularization (DRR) method, we ensure that the intrinsic structure of the drugs doesn’t change. Then, the final optimization objective of this work is obtained by combining feature learning and DRR using MDFLDRR. For Goal 1, the interaction score between drug *i* and drug *j* is represented by *S*_*ij*_, and the score directly reflects the likelihood of drug interaction. For Goal 2, if a drug with no known interactions generates a new interaction with drugs in the data set, we predict it through $J_{*}=\frac{\sum_{i=1}^{m}H_{*}^{i}Z^{i}}{m+1}$. Here, $H_{*}^{i}$ corresponds to the feature vector of this drug in *i*-th feature matrix.

## Materials and methods

In this section, we present the sources of the data sets used in the experiments, the learning process of MDFLDRR, the objective function and its corresponding solution, the description of drug molecular structure transformation, as well as the core pseudocode.

### Data set description

The following steps were taken to acquire and preprocess the data sets required for this work. First, access was granted from the Official DrugBank Online [37] to acquire drug information containing interaction, enzyme, target, and chemical substructure. Second, the id mapping system (https://www.uniprot.org/id-mapping) provided by the UniProt Database [38] was used to map the target proteins of the drugs to the KEGG DRUG Database [39] in order to acquire drug pathways. Third, drugs with no known interactions were removed, resulting in four different data sets that encompass interaction, pathway, enzyme, target, and chemical substructure.

The description of the data sets processed through the above operations is shown in Table 1. These drugs are described by four features: pathway, enzyme, target, and chemical substructure. Since each feature can be described by a set of corresponding descriptors, different binary feature vectors can be used to represent different drugs. If the corresponding descriptor exists, the value is set to 1; if not, the value defaults to 0. For instance, these different drugs can be described using molecular fingerprints to highlight their differences, and there are 881 substructure descriptors defined by PubChem [40]. Therefore, when describing the drug using the chemical substructure feature, an 881-dimensional feature vector can be used to represent the drug.

**Table 1:** Data sets utilized in this research.

Drug Data Set	Drug	Interaction	Pathway*	Enzyme*	Target*	Chemical Substructure*
*Data* *Set* 1	1854	165,439	430	294	656	881
*Data* *Set* 2	1278	90,286	272	185	425	
*Data* *Set* 3	2142	216,083	667	529	805	
*Data* *Set* 4	1566	125,308	306	221	481	

* represents drug features.

### Multi-Drug Features Learning with Drug Relation Regularization

#### Learning based on drug feature

Integrating multiple drug feature information not only improves prediction accuracy but also effectively describes different drugs. Since we have collected four different types of drug features, different drug features can be used to represent the same drug. Therefore, how to effectively combine different drug features to collectively represent the drug itself is a problem that needs to be considered.

Here, we consider mapping the feature vectors that can represent the drug itself based on different drug features into an interaction space using *H*^*i*^*Z*^*i*^, where the projection matrix is represented by *Z*^*i*^, the variable *i* takes values in the range [1, *m*]. Next, we let *H*^*i*^*Z*^*i*^ approximate to *S*, and use $||S-H^{i}Z^{i}{\left|\right|}_{F}^{2}+||J-H^{i}Z^{i}{\left|\right|}_{F}^{2}$ to represent the projection error, the variable *i* takes values in the range [1, *m*], where the Frobenius norm is represented by ||⋅||_*F*_. Simultaneously, to improve the generalization capability, we aim to control the sparsity of the projection matrix by attempting to minimize $\sum_{j=1}^{n}\left(|\right|Z_{\cdot j}^{i}{\left|\right|}^{2}+\left|\right|H_{\cdot j}^{i}{\left|\right|}^{2})$, where the *j*-th column of *Z*^*i*^ and *H*^*i*^ is denoted by $Z_{\cdot j}^{i}$ and $H_{\cdot j}^{i}$, respectively, the variables *i* and *j* take values in the range [1, *m*].

Based on the above content, we introduce tunable parameters *θ* and λ, and attempt to find a combination of *Z* and *S* that minimizes the objective equation defined as Eq (1), where *Z*^*i*^ ≥ 0 and *H*^*i*^ ≥ 0:

$$\begin{array}{c} \theta \sum_{i=1}^{m}(||S-H^{i}Z^{i}{\left|\right|}_{F}^{2}+||J-H^{i}Z^{i}{\left|\right|}_{F}^{2})+\lambda \sum_{i=1}^{m}\sum_{j=1}^{n}\left(|\right|Z_{\cdot j}^{i}{\left|\right|}^{2}+\left|\right|H_{\cdot j}^{i}{\left|\right|}^{2}{)+||S-J||}_{F}^{2} \end{array}$$ (1)

#### Learning based on known Drug-Drug Interaction

For the prediction of DDI, we base our approach on an assumption: unknown interactions share the same structure as known interactions. This implies that the structural patterns found among known DDIs may also exist in interactions that are still unknown or yet to be discovered. Therefore, we extract structural patterns from known DDIs and apply them in the prediction of new interactions. This approach leverages existing data resources to achieve more accurate results in subsequent DDI predictions.

In the interaction space, since every vector can represent a drug, each row in the interaction matrix *J*, constructed based on known DDIs, corresponds to a vector representing a drug. Therefore, we can reconstruct the vector for each drug using vectors representing different drugs, and attempt to find an *M* that minimizes the objective equation formulated as Eq (2), where *M* ≥ 0 and (*N* ⊙ *M*)*e* = *e*:

$$\begin{array}{c} \frac{1}{2}||\left(N⊙M\right)J-{J||}_{F}^{2}+\frac{1}{2}||NJ-{J||}^{2}+\frac{\theta }{2}\sum_{i=1}^{n}{||\left(N⊙M\right)}_{i\cdot }{\left|\right|}_{1}^{2} \end{array}$$ (2)

Here, the Hadamard product is denoted by the symbol ⊙, the 1-norm of the vector is represented by the symbol $\left|\right|\cdot {\left|\right|}_{1}^{2}$, the *i*-th row of *N* ⊙ *M* is denoted as (*N* ⊙ *M*)_*i*⋅_, a unit vector is represented by the symbol *e*. A boolean matrix of order *n* is denoted by the symbol *N*, where *N*_*ij*_ = 0 when *i* = *j* and 1 otherwise. If all *n* rows are considered, then the expression ${\left||\left(N⊙M\right)e|\right|}_{2}^{2}$ can be used to replace $\sum_{i=1}^{n}{||\left(N⊙M\right)}_{i\cdot }{\left|\right|}_{1}^{2}$ in Eq (2).

To clearly describe the structural form of known DDIs, we need to introduce the Lagrangian function as Eq (3) in order to compute *M*. Where the trace of a matrix is represented by *tr* and the Lagrange multiplier is represented by *φ*.

$$\begin{array}{c} L=\frac{1}{2}||\left(N⊙M\right)J-{J||}_{F}^{2}+\frac{1}{2}||NJ-{J||}^{2}+\frac{\theta }{2}{\left||\left(N⊙M\right)e|\right|}_{2}^{2}-tr\left(\varphi M\right)-\lambda^{T}\left(\left(N⊙M\right)e-e\right) \end{array}$$ (3)

After calculating the derivative of *L* with respect to *M* and applying the complementary slackness condition, the adjustment strategy for *M* is given by Eq (4):

$$\begin{array}{c} M_{ij}=\{\begin{array}{cccc} & 0, & & i=j\\ & M_{ij}\frac{{\left(\lambda ee^{T}\left(I+JJ^{T}\right)+JJ^{T}\right)}_{ij}}{{\left(\left(N⊙M\right)\left(\theta ee^{T}+\theta ee^{T}JJ^{T}+JJ^{T}\right)\right)}_{ij}}, & & otherwise \end{array} \end{array}$$ (4)

In order to ensure that the structural form of the drug will not change due to projection, $S_{i\cdot }-\sum_{l=1}^{n}M_{il}S_{l\cdot }$ should be close to zero, where the *i*-th row of *S* is expressed in the form of *S*_*i*⋅_ and the variable *i* takes values in the range [1, *n*]. Similarly, $S_{ij}-\sum_{l=1}^{n}M_{il}S_{lj}$ should also be close to zero, where the (*i*, *j*)-th item of *S* is expressed in the form of *S*_*ij*_ and the variables *i* and *j* take values in the range [1, *n*]. As before, we minimize the expression $S_{ij}-\sum_{l=1}^{n}M_{il}S_{lj}$. Ultimately, the defined Drug Relation Regularization (DRR) is as Eq (5):

$$\begin{array}{c} \sum_{i=1}^{n}\sum_{j=1}^{n}\left(S_{ij}-\sum_{l=1}^{n}M_{il}S_{lj}\right)\sum_{l=1}^{n}M_{il}S_{lj}=tr\left({\left(\left(I-M\right)S\right)}^{T}MS\right) \end{array}$$ (5)

#### Objective function and updating strategy

Based on the learning process of MDFLDRR elaborated in the previous two sections, we comprehensively consider Eqs (1) and (5), to determine the objective function of MDFLDRR, as shown in Eq (6), where *Z*^*i*^ ≥ 0 and *H*^*i*^ ≥ 0:

$$\begin{array}{cc} \theta \sum_{i=1}^{m}(||S-H^{i}Z^{i}{\left|\right|}_{F}^{2}+||J-H^{i}Z^{i}{\left|\right|}_{F}^{2}) & +\lambda \sum_{i=1}^{m}\sum_{j=1}^{n}(\left|\right|Z_{\cdot j}^{i}{\left|\right|}^{2}+\left|\right|H_{\cdot j}^{i}{\left|\right|}^{2})\\ & +||S-{J||}_{F}^{2}+\sigma \text{tr}({\left((I-M)S\right)}^{T}MS) \end{array}$$ (6)

Here, the sparsity of feature learning is affected by λ, the degree of deviation between the reconstruction matrix and the projection matrix is influenced by *θ*, and *σ* is the parameter used for performing DRR.

Let the partial derivative of Eq (6) with respect to *S* be zero, we have Eq (7):

$$\begin{array}{c} S={\left(\left(1+m\theta \right)I+\sigma {\left(I-M\right)}^{T}\right)}^{-1}\left(\theta \sum_{i=1}^{m}H^{i}Z^{i}+J\right) \end{array}$$ (7)

Here, for ease of subsequent description, we denote *Y* = ((1 + *mθ*)*I* + *σ*(*I* − *M*)^*T*^)^−1^. Next, we substitute Eq (7) into Eq (6) to obtain an objective function about ${\left\{Z^{i}\right\}}_{i=1}^{m}$, as shown in Eq (8):

$$\begin{array}{c} \begin{array}{cc} \sum_{i=1}^{m} & \left(\text{tr}(\lambda {\left(Z^{i}\right)}^{T}O_{n_{i}\times n_{i}}Z^{i}+\lambda {\left(H^{i}\right)}^{T}O_{n_{i}\times n_{i}}H^{i}\right)\\ & +\text{tr}(\theta {\left(Z^{i}\right)}^{T}{\left(H^{i}\right)}^{T}H^{i}Z^{i}-2\theta J^{T}YH^{i}Z^{i}))\\ & -\theta^{2}\sum_{i=1}^{m}\sum_{j=1}^{m}\text{tr}({\left(Z^{i}\right)}^{T}{\left(H^{i}\right)}^{T}Y^{T}H^{i}Z^{i}) \end{array} \end{array}$$ (8)

Here, the all-ones matrix is expressed by the symbol $O_{n_{i}\times n_{i}}$, where the number of columns in *H*^*i*^ is expressed by the symbol *n*_*i*_.

The updating strategy for *Z*^*i*^ is shown in Eq (9):

$$\begin{array}{c} Z_{ty}^{i}=Z_{ty}^{i}\sqrt{\frac{{\left(C^{-}Z^{i}+B^{+}\right)}_{ty}}{{\left(C^{+}Z^{i}+B^{-}\right)}_{ty}}} \end{array}$$ (9)

Here, $B^{+}=\frac{\left(|B|+B\right)}{2}$, $B^{-}=\frac{\left(|B|-B\right)}{2}$, $C^{+}=\frac{\left(|C|+C\right)}{2}$ and $C^{-}=\frac{\left(|C|-C\right)}{2}$.

#### Drug molecular structure transformation

**Fig 3:**
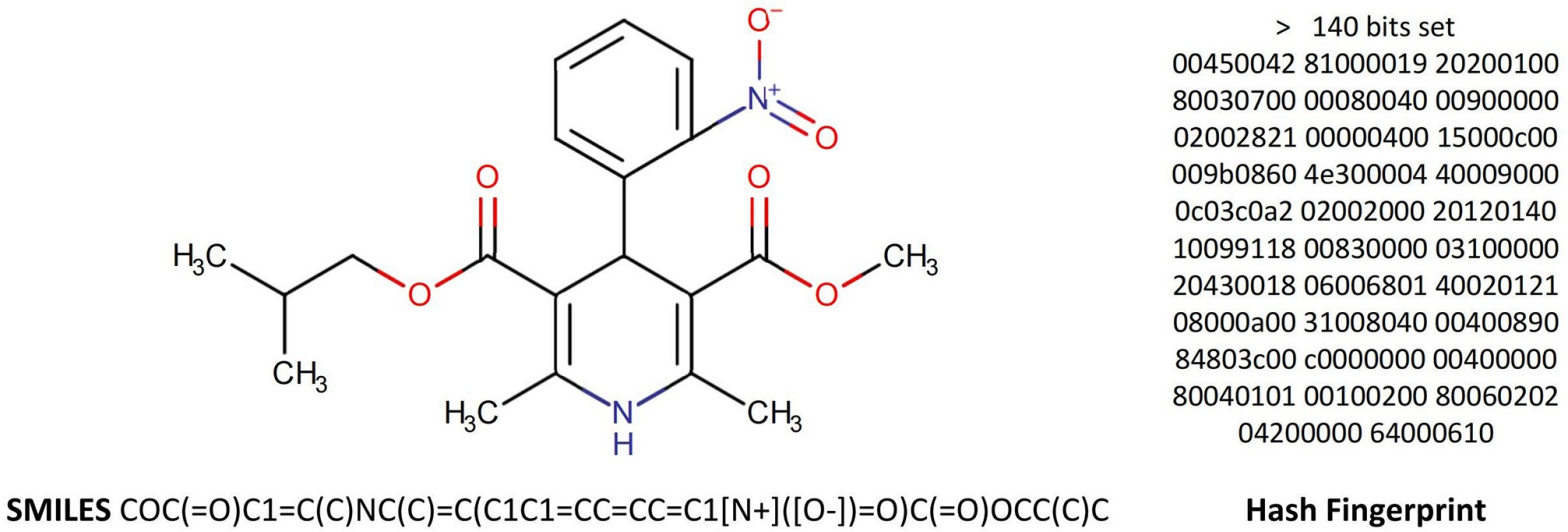


Simplified Molecular Input Line Entry System (SMILES) is a string representation method used to describe molecular structures. It employs ASCII characters to encode information about atoms, bonds, and rings, effectively transforming complex molecular structures into highly readable text form. The structure of a drug molecule refers to the arrangement of atoms and their chemical bonds within the molecule. By using SMILES notation, drug molecular structures can first be converted into SMILES strings and then transformed into hash fingerprints, allowing for computerized representation and processing of drug structures, as shown in Fig 3.

In this study, the calculation of the severity of drug interactions is based on the similarity of drug molecular structures. Simply put, if it is known that Drug A interacts with Drug B, then when Drug C is similar in molecular structure to Drug A (or Drug B), we have reason to believe that the higher the degree of similarity, the more likely Drug C is to interact with Drug B (or Drug A) and the severity would also be closer to the interaction severity between Drug A and Drug B. Therefore, by generating molecular fingerprints from SMILES representations of drug molecules, we can calculate structural similarity between these molecules to assess the severity level of potential drug interactions. Our method for computing Molecular Structure Similarity of Drugs (MSSD) is embodied in Eq (10).

$$\begin{array}{c} MSSD\left(A,B\right)=\alpha \cdot \frac{\left|A\cap B\right|}{\left|A\cup B\right|}+\beta \cdot \frac{2\cdot |A\cap B|}{\left|A|+|B\right|}+\left(1-\alpha -\beta \right)\cdot \frac{A^{\prime }\cdot B^{\prime }}{∥A^{\prime }∥\cdot ∥B^{\prime }∥} \end{array}$$ (10)

Specifically, *A* and *B* represent the fingerprints of two drug molecules, respectively. *A* ∩ *B* represents the intersection of the two fingerprints, and *A* ∪ *B* represents the union of the two fingerprints. *A*′ and *B*′ each denote the vector representations of the fingerprints of the two drug molecules. *A*′ ⋅ *B*′ represents the dot product of the two vectors, while ‖*A*′‖ and ‖*B*′‖ denote the magnitude of these vectors, respectively. *α* and *β* are parameters ranging from 0 to 1, which are set to 0.5 and 0.2 respectively in this research.

## Experiments and results

### Evaluation metrics

To balance the consumption of computing resources and more accurately reflect the model’s performance under different data distributions when evaluating machine learning model performance, we ultimately chose to use 5-fold Cross-Validation (5CV). For the two different DDI prediction goals that we proposed, we carried out 5CV separately for each of them.

**Fig 4:**
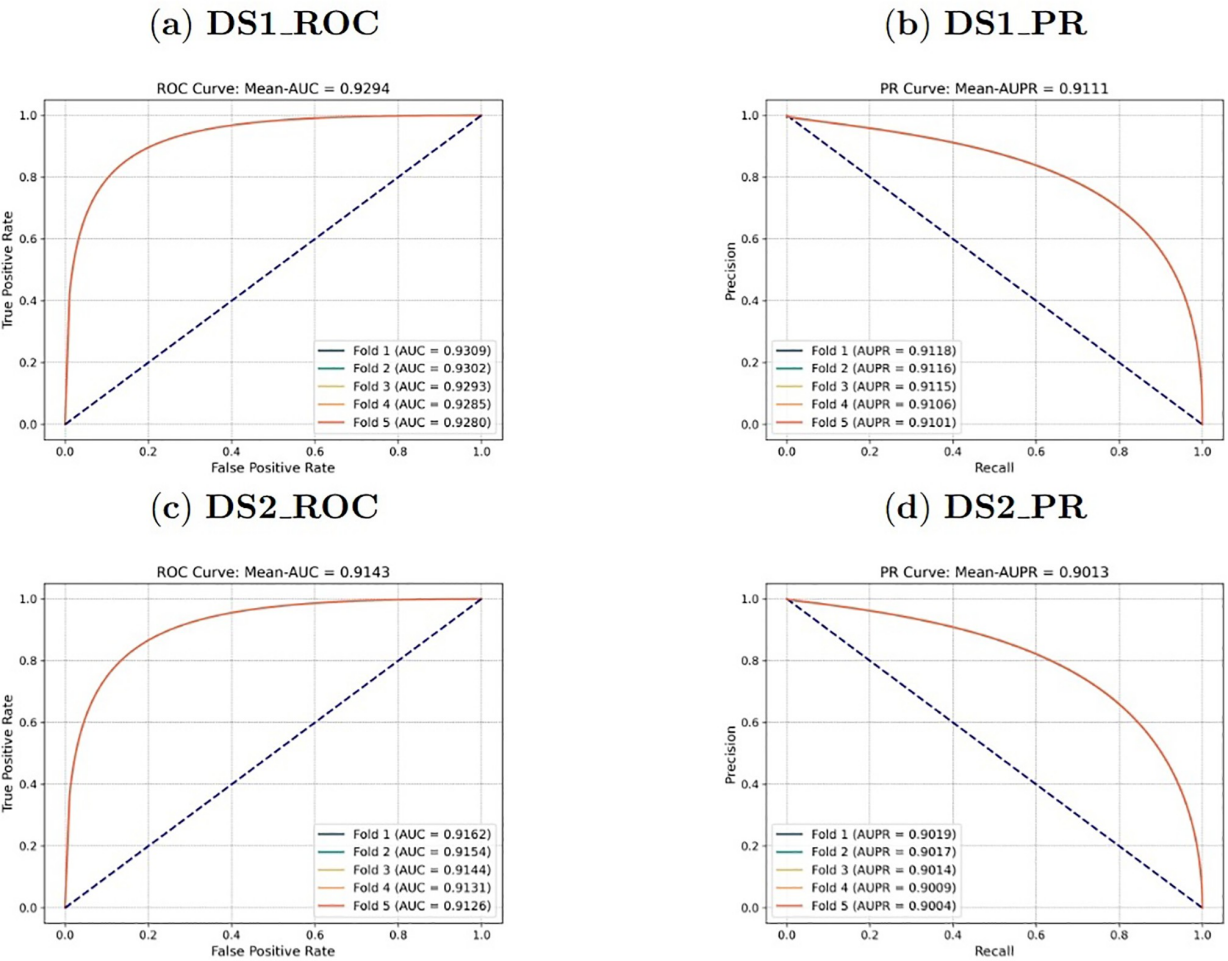


For Goal 1, the data set was randomly divided into five equal parts based on the existing interactions. For each iteration, 80% of the interaction data were selected as the training set, while the remaining 20% were used as the validation set. The model was then used to predict the DDIs that have not been observed yet among the drugs with existing interactions in the validation set, resulting in the curves shown in Fig 4.

**Fig 5:**
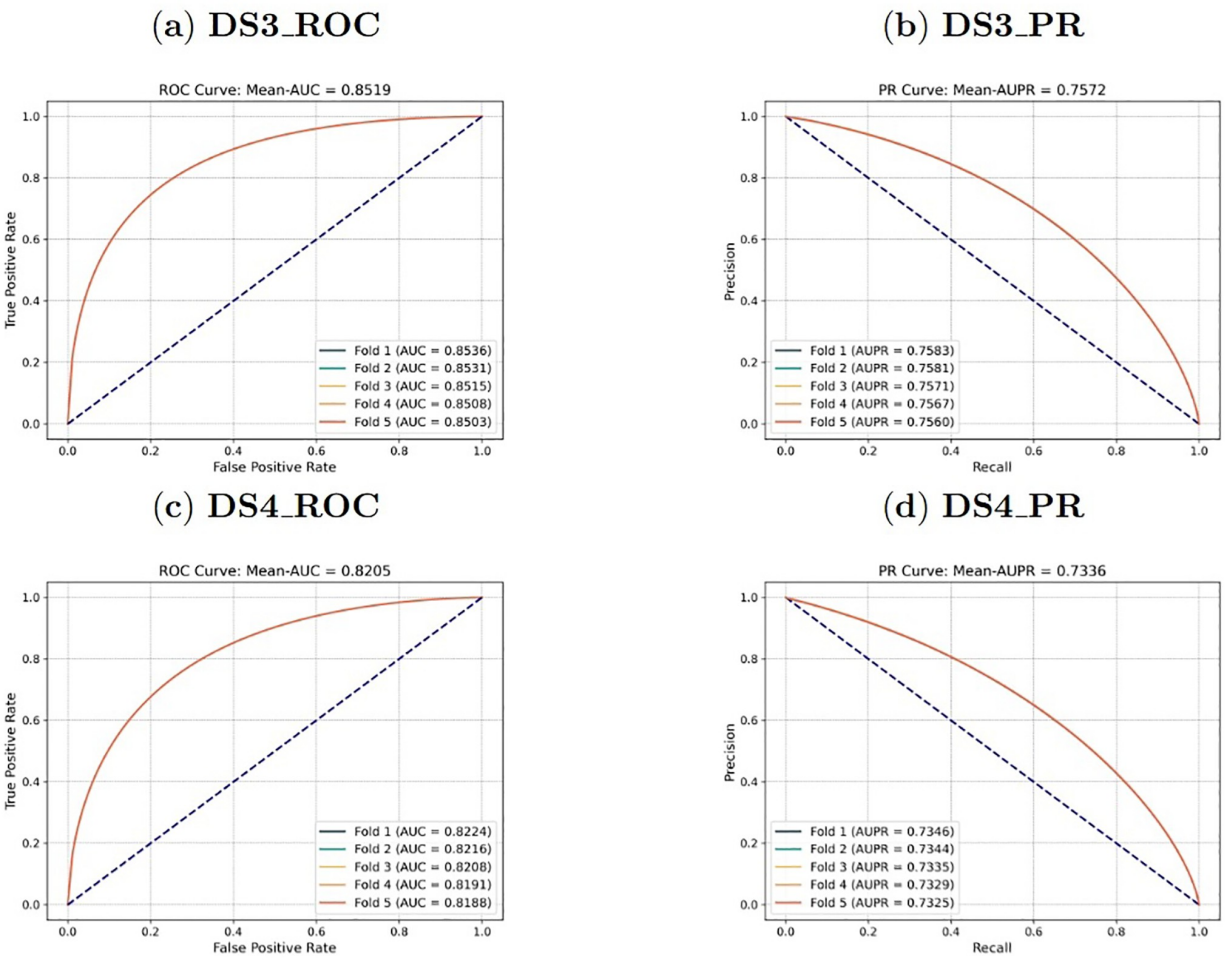


For Goal 2, all drugs were randomly divided into five equal parts. For each iteration, 80% of the drug data were used as the training set, while the remaining 20% were used as the validation set. The model was then used to predict the DDIs that have not been observed yet between known drugs inside the drug network and new drugs outside the network in the validation set, as shown in the curves depicted in Fig 5.

Six different metrics, including Recall, Precision, F_1_, Accuracy, Area Under the receiver operating characteristic Curve (AUC) and Area Under the Precision-Recall curve (AUPR), were employed to evaluate the prediction models, with their respective calculation methods given in Eqs (11) to (16). Moreover, AUPR was chosen as the primary metric for model performance evaluation for two reasons: First, a class imbalance exists in the DDI prediction task, where negative examples vastly outnumber positive ones. As a result, Accuracy isn’t a suitable primary evaluation metric. Instead, AUPR more accurately reflects the performance of the model in predicting positive examples, with a focus on Precision at high Recall rates. Second, AUPR calculation does not rely on a specific decision threshold. It can provide a comprehensive evaluation of the model’s performance at different Recall rates, making it adaptable to the varying Recall rate requirements in medical applications.

$$\begin{array}{c} \text{Recall}=\frac{\text{TP}}{\text{TP}+\text{FN}} \end{array}$$ (11)

$$\begin{array}{c} \text{Precision}=\frac{\text{TP}}{\text{TP}+\text{FP}} \end{array}$$ (12)

$$\begin{array}{c} {\text{F}}_{1}=2\times \frac{\text{Precision}\times \text{Recall}}{\text{Precision}+\text{Recall}} \end{array}$$ (13)

$$\begin{array}{c} \text{Accuracy}=\frac{\text{TP}+\text{TN}}{\text{TP}+\text{TN}+\text{FP}+\text{FN}} \end{array}$$ (14)

$$\begin{array}{c} \text{AUC}=\int \text{ROC}\;\text{Curve}\;d\text{FPR} \end{array}$$ (15)

$$\begin{array}{c} \text{AUPR}=\int \text{Precision-Recall}\;\text{Curve}\;d\text{Recall} \end{array}$$ (16)

Here, TP represents true positive, FN represents false negative, FP represents false positive, TN represents true negative, and FPR represents false positive rate, given by $\text{FPR}=\frac{\text{FP}}{\text{FP}+\text{TN}}$.

### Parameter sensitivity analysis

λ, *θ* and *σ* are the three tunable parameters of MDFLDRR, and the performance of the prediction model is directly affected by the values of these three parameters. In this work, values were selected from 10^−5^, 10^−4^, 10^−3^, 10^−2^ and 10^−1^ to assign to these three tunable parameters, and the model was tested using different combinations of these parameters.

**Fig 6:**
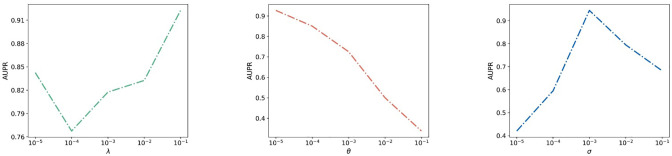


For Goal 1, the highest AUPR value was achieved by MDFLDRR when (λ, *θ*, *σ*) = (10^−1^, 10^−5^, 10^−3^). To facilitate the analysis of the impact of each tunable parameter on the performance of the prediction model, experiments were performed with two of the tunable parameters fixed, as shown in Fig 6. When λ and *θ* were fixed, although increasing the value of *σ* led to a decrease in the AUPR value after it reached its maximum, in the vast majority of cases, an increase in the value of *σ* was associated with an increase in the AUPR value. When λ and *σ* were fixed, the larger the value of *θ*, the smaller the AUPR value. When *θ* and *σ* were fixed, after the AUPR value dropped to its minimum, an increase in the value of λ brought about an increase in the AUPR value. Unless otherwise specified, the values of the tunable parameters used in the MDFLDRR in the subsequent comparative experiments concerning Goal 1 are assumed to be (λ, *θ*, *σ*) = (10^−1^, 10^−5^, 10^−3^).

**Fig 7:**
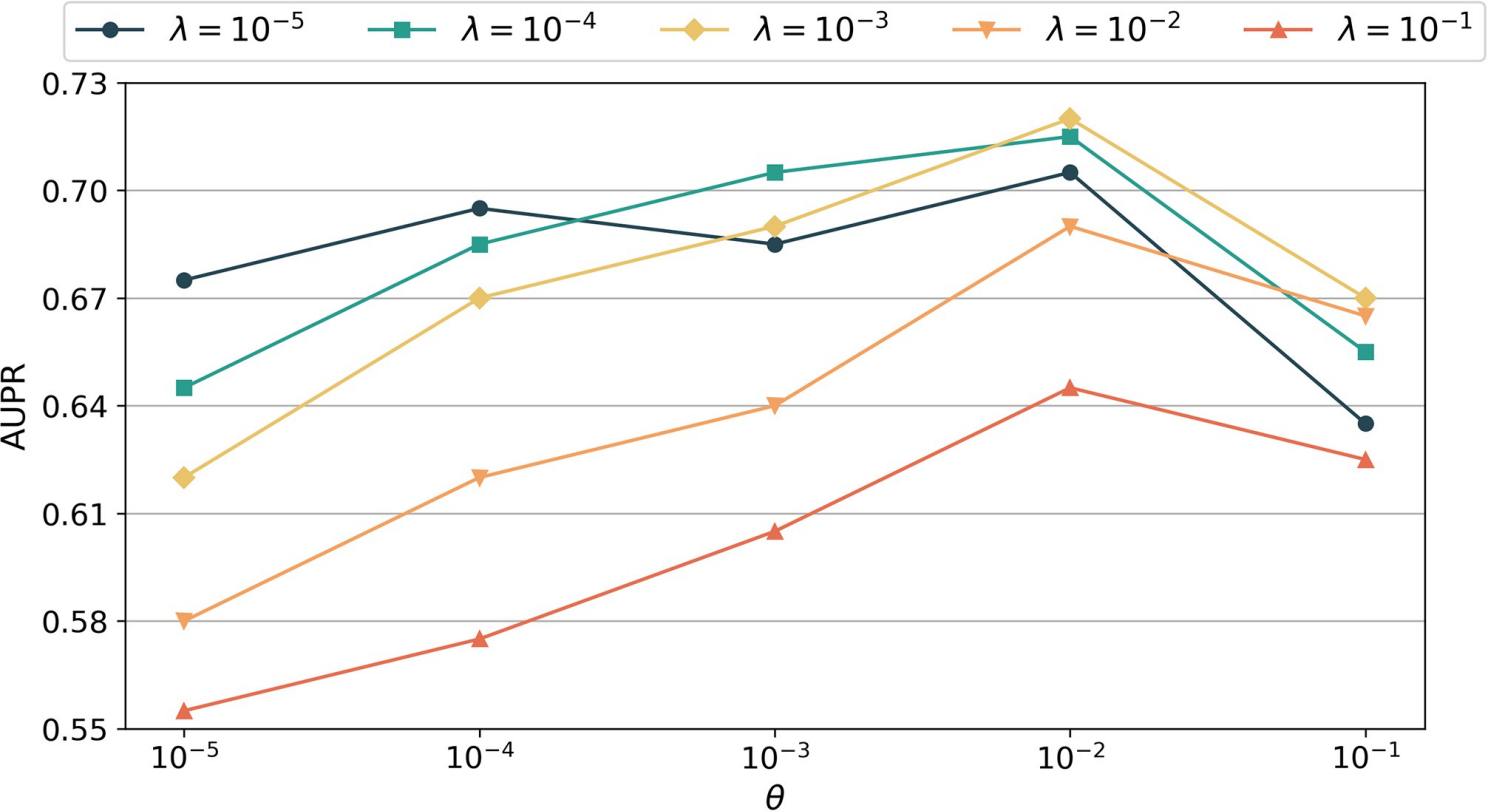


For Goal 2, since there are no connections between drugs without known interactions during DRR, we set the tunable parameter *σ* to 0. The experimental results, as depicted in Fig 7, demonstrate that the AUPR value is significantly influenced by the values of λ and *θ*, directly affecting the prediction results. Among the tested parameter combinations, MDFLDRR achieves the highest AUPR value when (λ, *θ*, *σ*) = (10^−3^, 10^−2^, 0). Unless otherwise specified, the values of the tunable parameters used in the MDFLDRR in the subsequent comparative experiments concerning Goal 2 are assumed to be (λ, *θ*, *σ*) = (10^−3^, 10^−2^, 0).

### Method comparison

In this section, we have selected five current advanced DDI prediction methods as references: MSEDDI [41], GNN-DDI [42], SmileGNN [43], ISCMF [44], and CLML [45]. Through a series of experiments, we compared these methods with the MDFLDRR proposed in this paper. Additionally, we modified the SmileGNN, ISCMF, and CLML algorithms to enable their implementation for Goal 2. For each comparison method, we conducted experiments using the parameters recommended or defaulted in the related literature to achieve optimal performance.

The core work of the paper is decomposed into two goals to be accomplished separately. The comparative experimental results for Goal 1 are shown in Table 2. From the results, it can be observed that MDFLDRR achieves the best results in all aspects except for Recall and Precision. Across two data sets, the method MDFLDRR consistently outperforms others, namely MSEDDI, GNN-DDI, SmileGNN, ISCMF, and CLML. On data set 1, MDFLDRR exhibits enhancements of 2.2%—6.5% in F_1_, 4.7%—9.2% in Accuracy, 2.2%—5.9% in AUC, and 1.2%—6.6% in AUPR. This trend continues onto data set 2, where MDFLDRR manifests improvements of 1.0%—6.0% in F_1_, 3.4%—8.6% in Accuracy, 4.2%—10.4% in AUC, and 1.9%—10.5% in AUPR when compared to the same methods.

**Table 2:** Different methods perform on Goal 1.

***Data* *Set* 1**						
**Method**	**Recall**	**Precision**	**F_1_**	**Accuracy**	**AUC**	**AUPR**
MDFLDRR	0.9095 ± 0.0029	0.9161 ± 0.0019	*0.9128 ± 0.0017*	*0.9236 ± 0.0013*	*0.9294 ± 0.0014*	*0.9111 ± 0.0007*
MSEDDI	0.8882 ± 0.0048	0.8965 ± 0.0032	0.8923 ± 0.0027	0.8802 ± 0.0025	0.9093 ± 0.0039	0.9003 ± 0.0045
GNN-DDI	*0.9232 ± 0.0035*	0.8353 ± 0.0027	0.8771 ± 0.0022	0.8501 ± 0.0016	0.8939 ± 0.0056	0.8835 ± 0.0032
SmileGNN	0.8408 ± 0.0061	0.8663 ± 0.0041	0.8534 ± 0.0018	0.8642 ± 0.0030	0.8889 ± 0.0042	0.8717 ± 0.0059
ISCMF	0.8242 ± 0.0057	*0.9274 ± 0.0052*	0.8728 ± 0.0029	0.8382 ± 0.0023	0.8855 ± 0.0051	0.8509 ± 0.0041
CLML	0.8384 ± 0.0072	0.8704 ± 0.0046	0.8541 ± 0.0030	0.8789 ± 0.0028	0.8747 ± 0.0045	0.8617 ± 0.0062
***Data* *Set* 2**						
**Method**	**Recall**	**Precision**	**F_1_**	**Accuracy**	**AUC**	**AUPR**
MDFLDRR	0.8849 ± 0.0053	0.8978 ± 0.0028	*0.8913 ± 0.0026*	*0.9059 ± 0.0041*	*0.9143 ± 0.0017*	*0.9013 ± 0.0006*
MSEDDI	0.8527 ± 0.0068	*0.9148 ± 0.0035*	0.8827 ± 0.0038	0.8750 ± 0.0045	0.8756 ± 0.0032	0.8842 ± 0.0029
GNN-DDI	0.8955 ± 0.0062	0.8418 ± 0.0057	0.8678 ± 0.0044	0.8646 ± 0.0035	0.8624 ± 0.0027	0.8505 ± 0.0042
SmileGNN	*0.9072 ± 0.0052*	0.8169 ± 0.0023	0.8597 ± 0.0033	0.8575 ± 0.0047	0.8478 ± 0.0048	0.8237 ± 0.0044
ISCMF	0.8334 ± 0.0043	0.8711 ± 0.0059	0.8518 ± 0.0042	0.8279 ± 0.0033	0.8191 ± 0.0038	0.8065 ± 0.0036
CLML	0.8213 ± 0.0074	0.8548 ± 0.0039	0.8377 ± 0.0041	0.8498 ± 0.0043	0.8277 ± 0.0028	0.8366 ± 0.0031

NOTE: The maximum value in each metric is italic.

The comparative experimental results for Goal 2 are shown in Table 3. From the results, it can be observed that MDFLDRR ranks second in terms of Recall and Precision, and first in all other aspects. Compared to the baseline algorithms, MDFLDRR indicates improvements of 2.8%—12.5% in F_1_, 6.9%—16.4% in Accuracy, 10.0%—19.6% in AUC, and 10.2%—19.4% in AUPR on data set 3. Similarly, MDFLDRR shows enhancements of 4.5%—13.9% in F_1_, 8.1%—17.6% in Accuracy, 11.1%—20.6% in AUC, and 11.2%—20.4% in AUPR on data set 4.

**Table 3:** Different methods perform on Goal 2.

***Data* *Set* 3**						
**Method**	**Recall**	**Precision**	**F_1_**	**Accuracy**	**AUC**	**AUPR**
MDFLDRR	0.8528 ± 0.0038	0.8094 ± 0.0021	*0.8305 ± 0.0020*	*0.8300 ± 0.0022*	*0.8519 ± 0.0016*	*0.7572 ± 0.0011*
MSEDDI	0.7984 ± 0.0045	*0.8171 ± 0.0038*	0.8076 ± 0.0026	0.7728 ± 0.0027	0.7589 ± 0.0035	0.6654 ± 0.0025
GNN-DDI	0.7739 ± 0.0062	0.7688 ± 0.0025	0.7713 ± 0.0023	0.7310 ± 0.0031	0.7437 ± 0.0041	0.6360 ± 0.0049
SmileGNN	0.7561 ± 0.0052	0.7794 ± 0.0035	0.7676 ± 0.0028	0.7463 ± 0.0019	0.7392 ± 0.0029	0.6450 ± 0.0036
ISCMF	0.7223 ± 0.0069	0.7316 ± 0.0057	0.7269 ± 0.0021	0.6941 ± 0.0033	0.6853 ± 0.0043	0.6101 ± 0.0052
CLML	*0.8691 ± 0.0041*	0.7251 ± 0.0049	0.7906 ± 0.0031	0.7537 ± 0.0020	0.7669 ± 0.0053	0.6800 ± 0.0056
***Data* *Set* 4**						
**Method**	**Recall**	**Precision**	**F_1_**	**Accuracy**	**AUC**	**AUPR**
MDFLDRR	0.8175 ± 0.0048	0.7811 ± 0.0043	*0.7989 ± 0.0029*	*0.7968 ± 0.0026*	*0.8205 ± 0.0017*	*0.7336 ± 0.0010*
MSEDDI	0.7744 ± 0.0046	0.7517 ± 0.0039	0.7629 ± 0.0032	0.7319 ± 0.0030	0.7221 ± 0.0045	0.6376 ± 0.0022
GNN-DDI	*0.8220 ± 0.0035*	0.7049 ± 0.0054	0.7590 ± 0.0030	0.7064 ± 0.0029	0.7031 ± 0.0032	0.6178 ± 0.0038
SmileGNN	0.7134 ± 0.0053	*0.7921 ± 0.0037*	0.7507 ± 0.0018	0.6918 ± 0.0021	0.7074 ± 0.0023	0.6090 ± 0.0043
ISCMF	0.6806 ± 0.0058	0.6960 ± 0.0048	0.6882 ± 0.0034	0.6563 ± 0.0018	0.6511 ± 0.0047	0.5838 ± 0.0030
CLML	0.8030 ± 0.0049	0.6997 ± 0.0028	0.7478 ± 0.0017	0.7136 ± 0.0034	0.7298 ± 0.0051	0.6518 ± 0.0020

NOTE: The maximum value in each metric is italic.

Based on previous experimental analysis, we obtained the performance of MDFLDRR combining four different drug features for achieving Goal 1 and Goal 2. To evaluate the quality of each drug feature learned by MDFLDRR, we separately used MDFLDRR with a single drug feature to accomplish Goal 1 and Goal 2.

**Fig 8:**
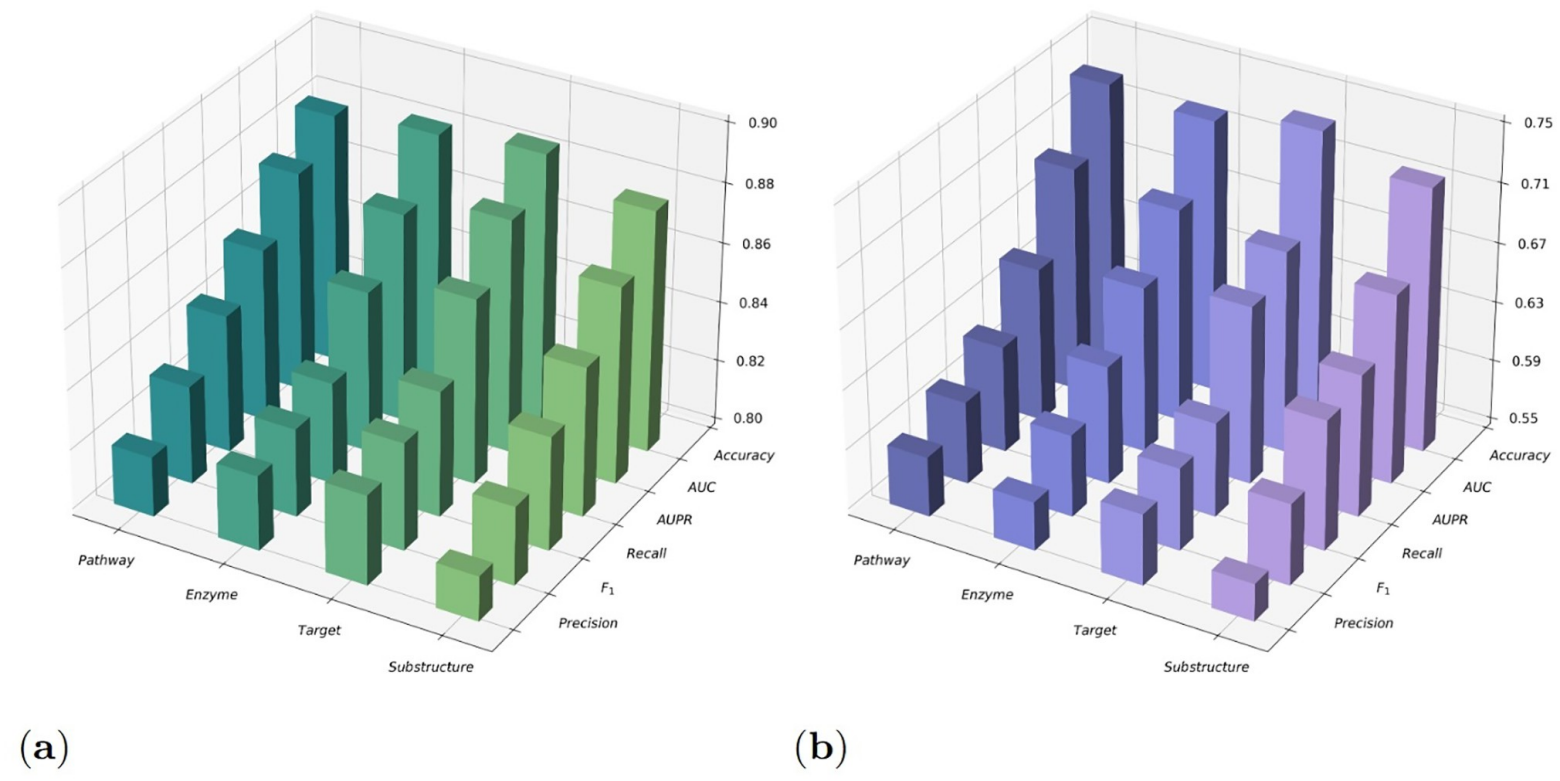


For Goal 1, compared to using only pathway, enzyme, target, and chemical substructure on data set 1, simultaneously learning all four drug features leads to improvements of 9.4%—11.0% in Precision, 8.4%—9.5% in F_1_, 7.0%—8.3% in Recall, 5.4%—6.7% in AUPR, 5.5%—6.8% in AUC, and 3.6%—4.6% in Accuracy, as depicted in Fig 8(a). Similarly, for Goal 2 on data set 3, compared to using only pathway, enzyme, target, and chemical substructure, simultaneously learning all four drug features leads to improvements of 26.2%—29.0% in Precision, 27.1%—27.2% in F_1_, 25.3%—28.1% in Recall, 11.6%—14.8% in AUPR, 17.7%—20.5% in AUC, and 10.2%—12.4% in Accuracy, as depicted in Fig 8(b). Although the improvements in certain metrics may not be significant, it indicates that the quality of each drug feature data we sought is sufficiently high.

### Case study

Typically, there are three possible severity levels, namely MINOR, MODERATE and MAJOR. By using the drug interaction checkers provided by DrugBank Online (https://go.drugbank.com/drug-interaction-checker) and Drugs.com (https://www.drugs.com/drug_interactions.html), we obtained the severity level for all predicted DDIs that have records in the databases. In order to provide a definitive severity level for DDIs that are not currently recorded in the drug databases, we came up with the idea of using the similarity of drug molecular structures to achieve this purpose. Since we can only obtain severity level from the drug databases, it is necessary to convert them into numerical values for calculation. As a result, we obtained the corresponding relationship as shown in Table 4.

**Table 4:** Severity score criteria.

Severity Score	Severity Level
1–30	MINOR (MI)
31–60	MODERATE (MO)
61–100	MAJOR (MA)

**Fig 9:**
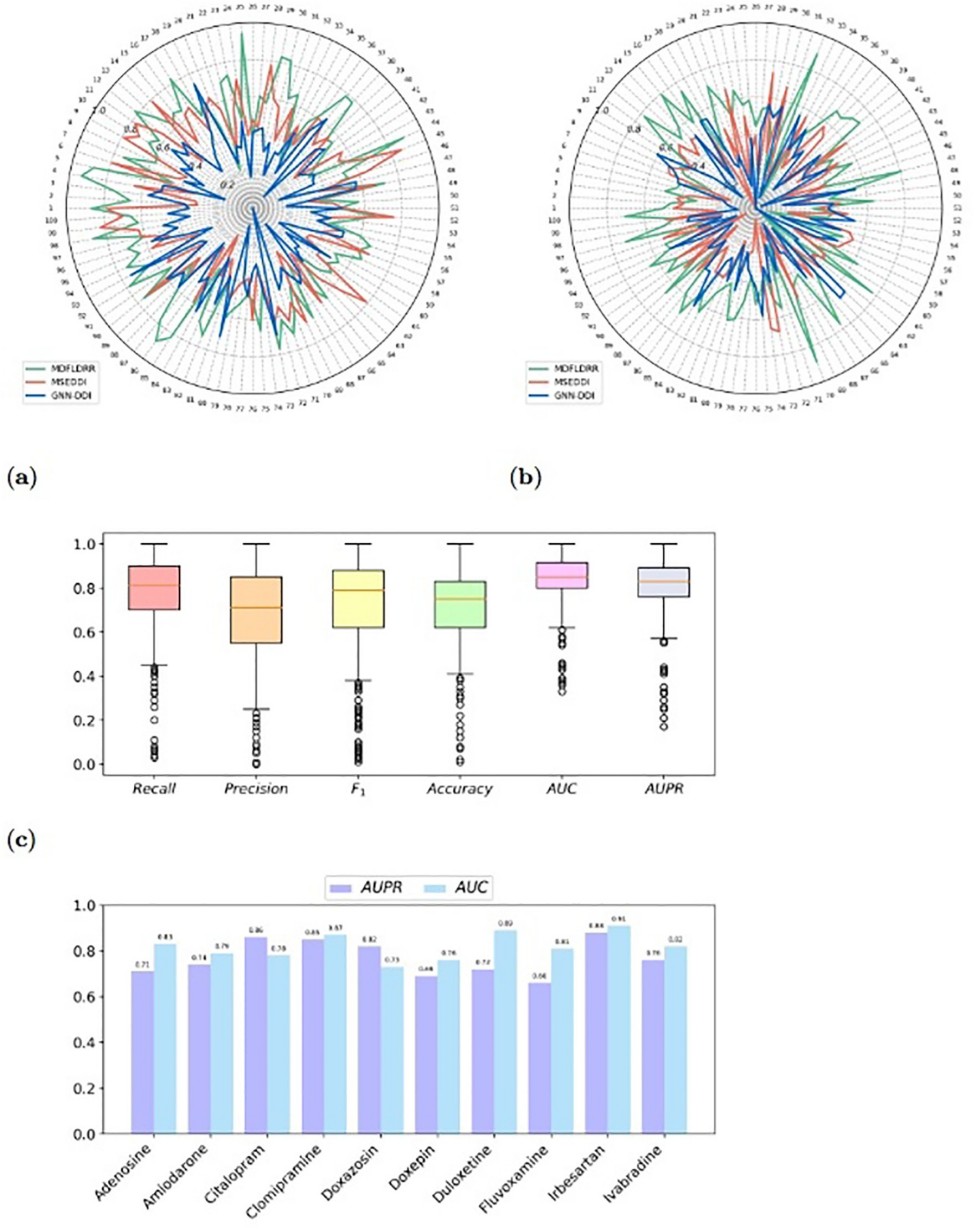


After discussing the categorization of severity levels and introducing the severity score proposed by us, we examine the performance of MDFLDRR in predicting DDIs between drugs from the training set and the test set. Firstly, we selected three algorithms that performed well overall in Section, to test the AUPR values of the same 100 pairs of DDIs predicted by different models. Among these, during the accomplishment of Goal 1, the AUPR values for the 100 pairs of DDIs on the combined data set of DS1 and DS2 are displayed in Fig 9(a); during the achievement of Goal 2, the AUPR values for the DDIs on the combined data set of DS3 and DS4 are depicted in Fig 9(b). Secondly, by using 66.67% of the drugs as the training set to simulate drugs with known DDIs, performing hyperparameter tuning and overfitting verification on the validation set consisting of 16.67% of drugs, and the remaining drugs as the test set to simulate drugs without known DDIs, we conducted an analysis of the experimental data and summarized the overall scores of the test set drugs, as shown in Fig 9(c). Notably, for the majority of test set drugs, MDFLDRR achieved high AUC and AUPR values, and the first quartile, third quartile, and median demonstrate the robustness and excellent prediction ability of MDFLDRR. Lastly, Fig 9(d) illustrates the performance of MDFLDRR on five randomly selected cardiovascular drugs and five antidepressants from the test set. In conclusion, the findings from the case study provide direct evidence that MDFLDRR is capable of accurately predicting interactions for drugs that lack established knowledge of DDIs.

**Fig 10:**
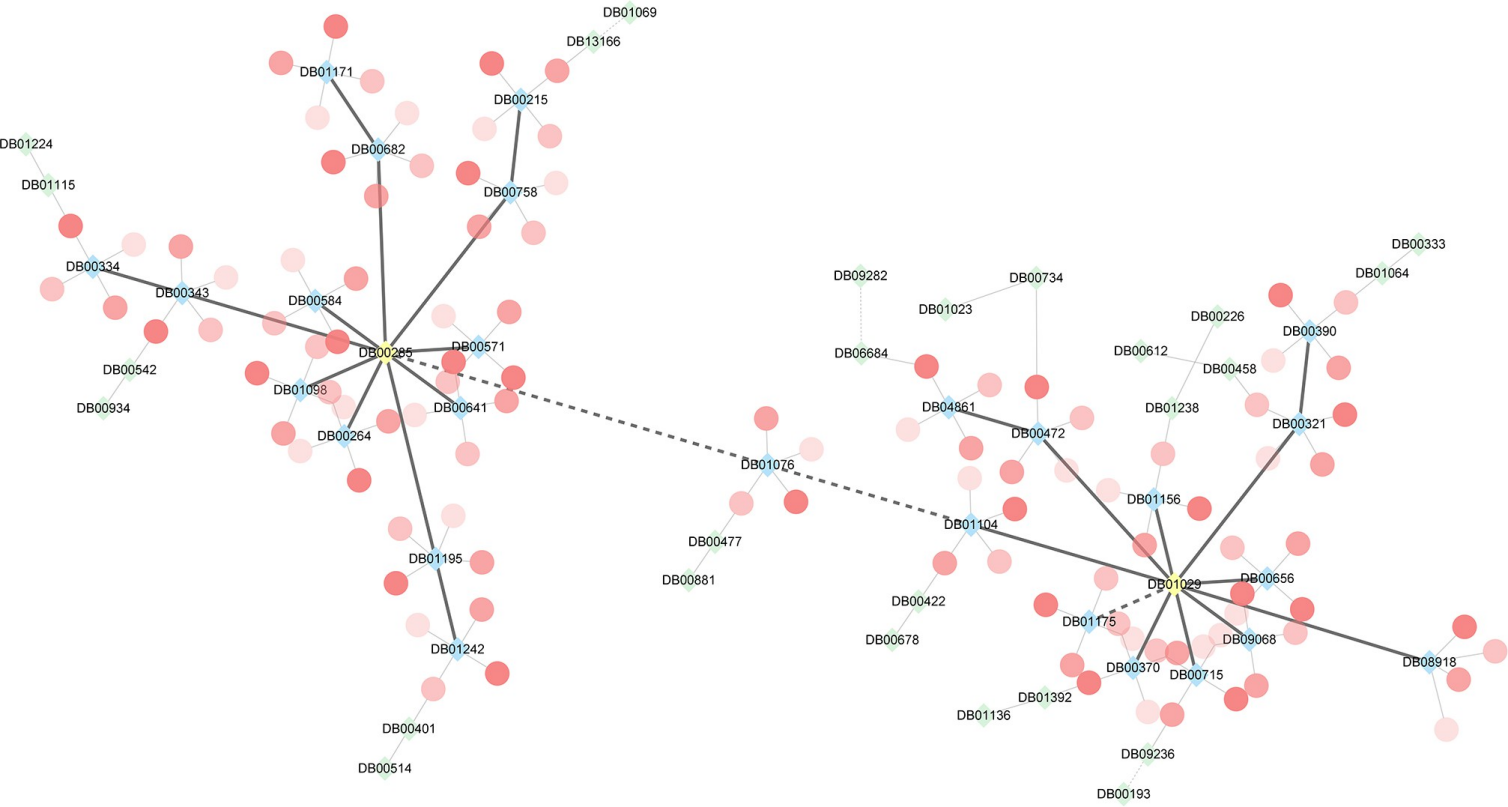


*Irbesartan* (DB01029) is a widely used medication in clinical practice. Meanwhile, *Venlafaxine* (DB00285) is an antidepressant drug primarily prescribed for treating major depressive disorder and other mental disorders. To further demonstrate the reliability of MDFLDRR, we conducted dual validation of the predicted antidepressants that interact with *Irbesartan* using DrugBank Online and Drugs.com, as well as cardiovascular drugs that interact with *Venlafaxine*. The results can be seen in Tables 5 and 6. Additionally, we provided the top 60 ranked interactions between cardiovascular drugs and antidepressants predicted by MDFLDRR, as depicted in Table 7. Noteworthily, the severity level obtained through drug databases and the severity score calculated by us are also presented in these tables for easy cross-checking. Lastly, due to space limitations and for a clearer observation, we only visualized the 40 pairs of DDIs mentioned in this paper and did not display all the DDIs predicted in this research, as depicted in Fig 10.

**Table 5:** Top 10 ranked antidepressants based on the intensity of their interactions with *Irbesartan*.

DrugBank ID	Drug	Chemical Structure (Fig 11)	Severity Level
DB00715	Paroxetine	(a1)	40*‖MO^0^^,^ ^1^
DB00656	Trazodone	(a2)	22*‖MI^0^^,^ ^1^‖MO^2^
DB08918	Levomilnacipran	(a3)	23*‖MI^0^‖MO^1^
DB09068	Vortioxetine	(a4)	27*‖MI^0^‖MO^1^
DB00472	Fluoxetine	(a5)	46*‖MO^0^^,^ ^1^
DB01156	Bupropion	(a6)	32*‖MI^1^‖MO^0^^,^ ^2^
DB01104	Sertraline	(a7)	51*‖MO^0^^,^ ^1^
DB00321	Amitriptyline	(a8)	55*‖MO^0^^,^ ^1^^,^ ^2^
DB00370	Mirtazapine	(a9)	28*‖MI^0^^,^ ^1^‖MO^2^
DB01175	Escitalopram	(a10)	17*‖MI^0^(*N*.*A*.)

* represents the severity score calculated by MDFLDRR,

^0^ represents the severity level determined by MDFLDRR,

^1^ represents evidence sourced from DrugBank Online,

^2^ represents evidence sourced from Drugs.com, (*N*.*A*.) stands for no public interaction records available.

**Table 6:** Top 10 ranked cardiovascular drugs based on the intensity of their interactions with *Venlafaxine*.

DrugBank ID	Drug	Chemical Structure(Fig 12)	Severity Level
DB00682	Warfarin	(b1)	20*‖MI^0^^,^^1^‖MO^2^
DB01098	Rosuvastatin	(b2)	38*‖MI^1^‖MO^0^
DB00343	Diltiazem	(b3)	49*‖MO^0^^,^^1^^,^^2^
DB00584	Enalapril	(b4)	23*‖MI^0^(*N*.*A*.)
DB00758	Clopidogrel	(b5)	34*‖MI^1^‖MO^0^^,^^2^
DB00571	Propranolol	(b6)	42*‖MO^0^^,^^1^
DB00641	Simvastatin	(b7)	78*‖MO^1^‖MA^0^
DB01195	Flecainide	(b8)	54*‖MO^0^^,^^1^^,^^2^
DB01076	Atorvastatin	(b9)	66*‖MA^0^(*N*.*A*.)
DB00264	Metoprolol	(b10)	31*‖MI^1^‖MO^0^

* represents the severity score calculated by MDFLDRR,

^0^ represents the severity level determined by MDFLDRR,

^1^ represents evidence sourced from DrugBank Online,

^2^ represents evidence sourced from Drugs.com, (*N*.*A*.) stands for no public interaction records available.

**Table 7:** Top 60 ranked interactions in intensity between cardiovascular drugs and antidepressants predicted by MDFLDRR.

DrugBank ID A	DrugBank ID B	Drug A	Drug B	Severity Level
DB01076	DB01104	Atorvastatin	Sertraline	17*‖MI^0^(*N*.*A*.)
DB00881	DB00477	Quinapril	Chlorpromazine	24*‖MI^0^^,^^1^‖MO^2^
DB00682	DB01171	Warfarin	Moclobemide	19*‖MI^0^‖MO^1^
DB01115	DB01224	Nifedipine	Quetiapine	37*‖MO^0^^,^^1^^,^^2^
DB09282	DB06684	Molsidomine	Vilazodone	62*‖MA^0^(*N*.*A*.)
DB01136	DB01392	Carvedilol	Yohimbine	36*‖MI^1^‖MO^0^
DB00612	DB00458	Bisoprolol	Imipramine	31*‖MO^0^^,^^1^^,^^2^
DB00390	DB00321	Digoxin	Amitriptyline	20*‖MI^0^‖MO^1^
DB01023	DB00734	Felodipine	Risperidone	32*‖MI^1^‖MO^0^^,^^2^
DB00226	DB01238	Guanadrel	Aripiprazole	21*‖MI^0^‖MO^2^
DB00678	DB00422	Losartan	Methylphenidate	48*‖MI^1^‖MO^0^^,^^2^
DB01195	DB01242	Flecainide	Clomipramine	70*‖MO^2^‖MA^0^^,^^1^
DB01064	DB00333	Isoproterenol	Methadone	83*‖MA^0^^,^^2^
DB00343	DB00334	Diltiazem	Olanzapine	42*‖MI^1^‖MO^0^^,^^2^
DB13166	DB01069	Zofenopril	Promethazine	57*‖MO^0^(*N*.*A*.)
DB00542	DB00934	Benazepril	Maprotiline	35*‖MO^0^^,^^2^
DB04861	DB00472	Nebivolol	Fluoxetine	54*‖MO^0^^,^^1^^,^^2^
DB09236	DB00193	Lacidipine	Tramadol	41*‖MO^0^(*N*.*A*.)
DB00401	DB00514	Nisoldipine	Dextromethorphan	13*‖MI^0^^,^^1^
DB00758	DB00215	Clopidogrel	Citalopram	46*‖MO^0^^,^^1^^,^^2^
DB00381	DB01104	Amlodipine	Sertraline	50*‖MO^0^^,^^1^
DB00727	DB01142	Nitroglycerin	Doxepin	20*‖MI^0^^,^^1^‖MO^2^
DB01023	DB00472	Felodipine	Fluoxetine	38*‖MO^0^^,^^1^^,^^2^
DB01136	DB01175	Carvedilol	Escitalopram	31*‖MO^0^^,^^1^
DB00819	DB00176	Acetazolamide	Fluvoxamine	37*‖MO^0^^,^^1^^,^^2^
DB00388	DB00842	Phenylephrine	Oxazepam	49*‖MO^0^^,^^1^
DB00388	DB00176	Phenylephrine	Fluvoxamine	33*‖MO^0^^,^^1^
DB01364	DB00472	Ephedrine	Fluoxetine	35*‖MO^0^(*N*.*A*.)
DB00281	DB00370	Lidocaine	Mirtazapine	43*‖MO^0^^,^^1^
DB01076	DB00252	Atorvastatin	Phenytoin	41*‖MO^0^^,^^1^^,^^2^
DB01109	DB01175	Heparin	Escitalopram	54*‖MO^0^^,^^1^^,^^2^
DB01002	DB00176	Levobupivacaine	Fluvoxamine	66*‖MO^2^‖MA^0^^,^^1^
DB00876	DB00824	Eprosartan	Oxazepam	44*‖MO^0^^,^^2^
DB00594	DB01175	Amiloride	Escitalopram	15*‖MI^0^‖MO^2^
DB08816	DB01104	Ticagrelor	Sertraline	51*‖MO^0^^,^^1^^,^^2^
DB00883	DB00176	Isosorbide dinitrate	Fluvoxamine	10*‖MI^0^(*N*.*A*.)
DB13919	DB01142	Candesartan	Doxepin	18*‖MI^0^^,^^1^‖MO^2^
DB06228	DB00472	Rivaroxaban	Fluoxetine	52*‖MO^0^^,^^1^^,^^2^
DB01002	DB09068	Levobupivacaine	Vortioxetine	27*‖MI^0^‖MO^1^
DB00388	DB01175	Phenylephrine	Escitalopram	36*‖MO^0^(*N*.*A*.)
DB00912	DB01142	Repaglinide	Doxepin	23*‖MI^0^‖MO^1^
DB00722	DB01175	Lisinopril	Escitalopram	7*‖MI^0^(*N*.*A*.)
DB00682	DB00715	Warfarin	Paroxetine	55*‖MO^0^^,^^1^^,^^2^
DB00264	DB00215	Metoprolol	Citalopram	41*‖MO^0^^,^^1^^,^^2^
DB00390	DB01224	Digoxin	Quetiapine	17*‖MI^0^‖MO^1^
DB00695	DB00476	Furosemide	Duloxetine	39*‖MI^1^‖MO^0^^,^^2^
DB00264	DB00472	Warfarin	Fluoxetine	48*‖MO^0^^,^^1^^,^^2^
DB00661	DB01104	Verapamil	Sertraline	36*‖MO^0^^,^^1^
DB00264	DB00458	Metoprolol	Imipramine	46*‖MO^0^^,^^1^^,^^2^
DB00335	DB01142	Atenolol	Doxepin	42*‖MO^0^^,^^1^^,^^2^
DB00999	DB01156	Hydrochlorothiazide	Bupropion	39*‖MO^0^^,^^1^^,^^2^
DB00571	DB00715	Propranolol	Paroxetine	14*‖MI^0^‖MO^2^
DB00908	DB00540	Quinidine	Nortriptyline	73*‖MO^2^‖MA^0^^,^^1^
DB00682	DB01156	Warfarin	Bupropion	45*‖MO^0^^,^^1^^,^^2^
DB00177	DB01175	Valsartan	Escitalopram	37*‖MO^0^(*N*.*A*.)
DB00727	DB00780	Nitroglycerin	Phenelzine	47*‖MO^0^^,^^1^^,^^2^
DB00695	DB01175	Furosemide	Escitalopram	11*‖MI^0^‖MO^2^
DB00908	DB00734	Quinidine	Risperidone	82*‖MA^0^^,^^1^^,^^2^
DB00678	DB01151	Losartan	Desipramine	33*‖MI^1^‖MO^0^
DB00695	DB09128	Furosemide	Brexpiprazole	31*‖MI^1^‖MO^0^^,^^2^

Drug A represents a cardiovascular drug, Drug B represents an antidepressant.

* represents the severity score calculated by MDFLDRR,

^0^ represents the severity level determined by MDFLDRR,

^1^ represents evidence sourced from DrugBank Online,

^2^ represents evidence sourced from Drugs.com, (*N*.*A*.) stands for no public interaction records available.

**Fig 11:**
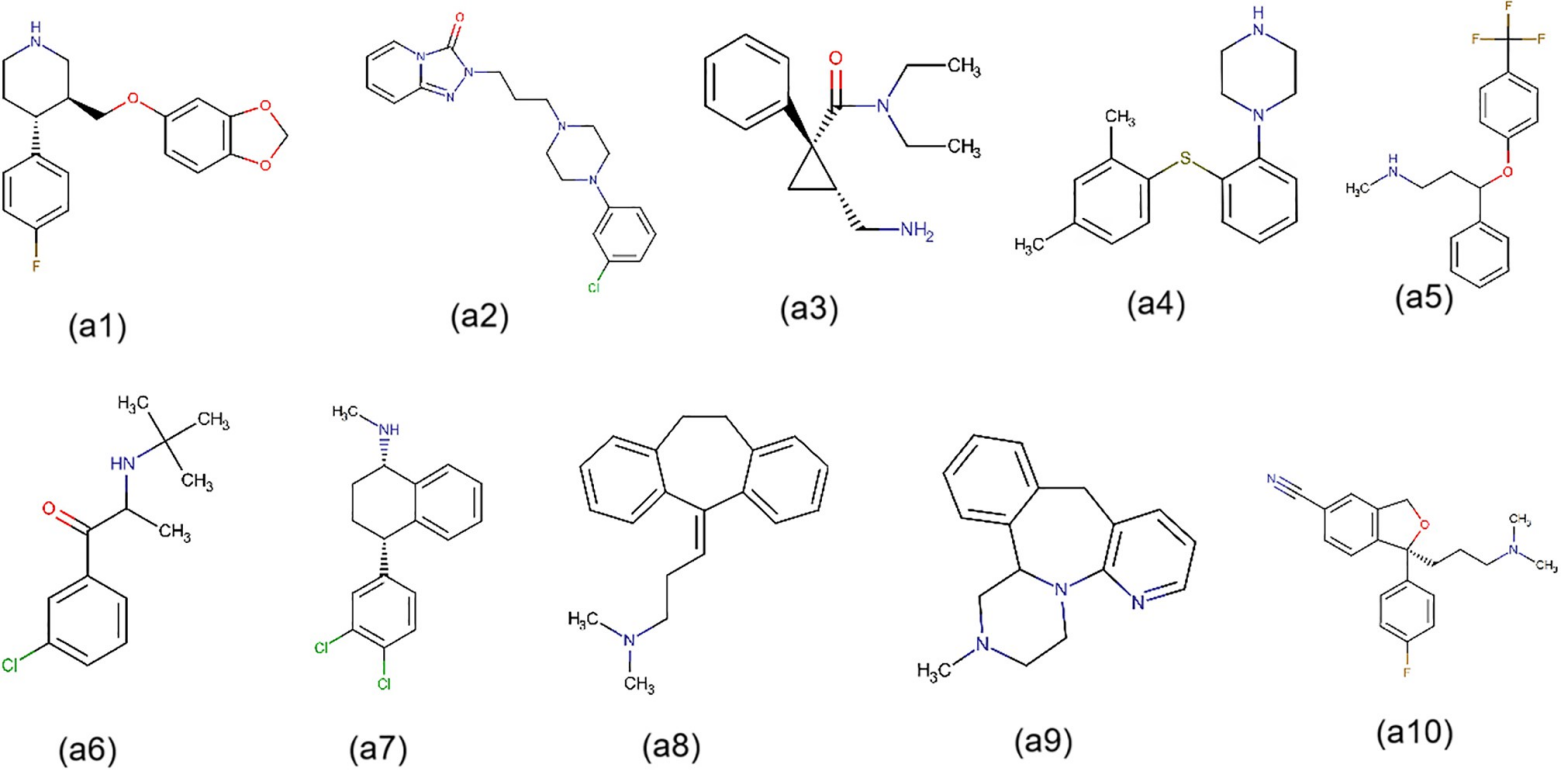


**Fig 12:**
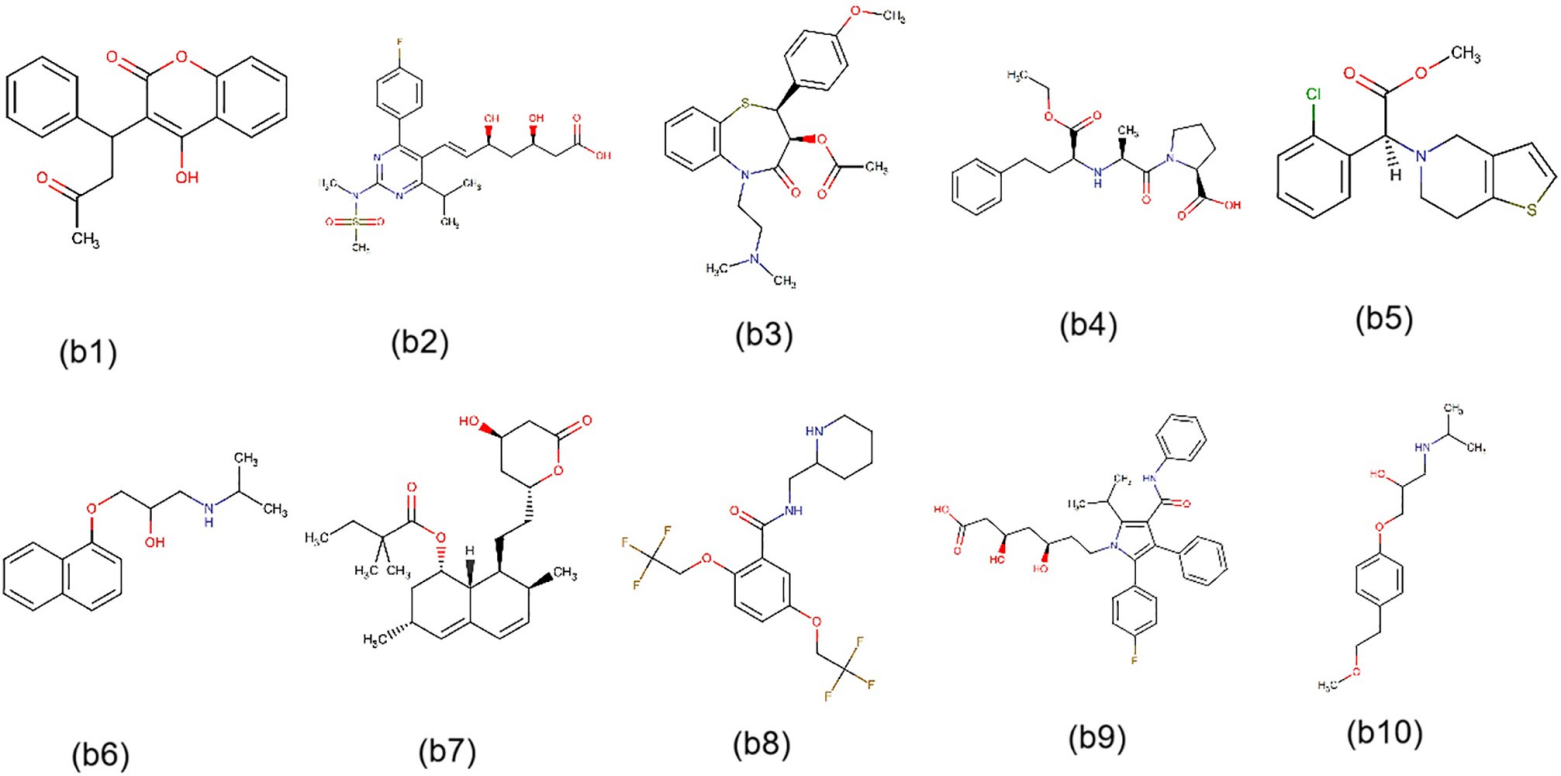


## Discussion

### Drug interaction severity level analysis

**Fig 13:**
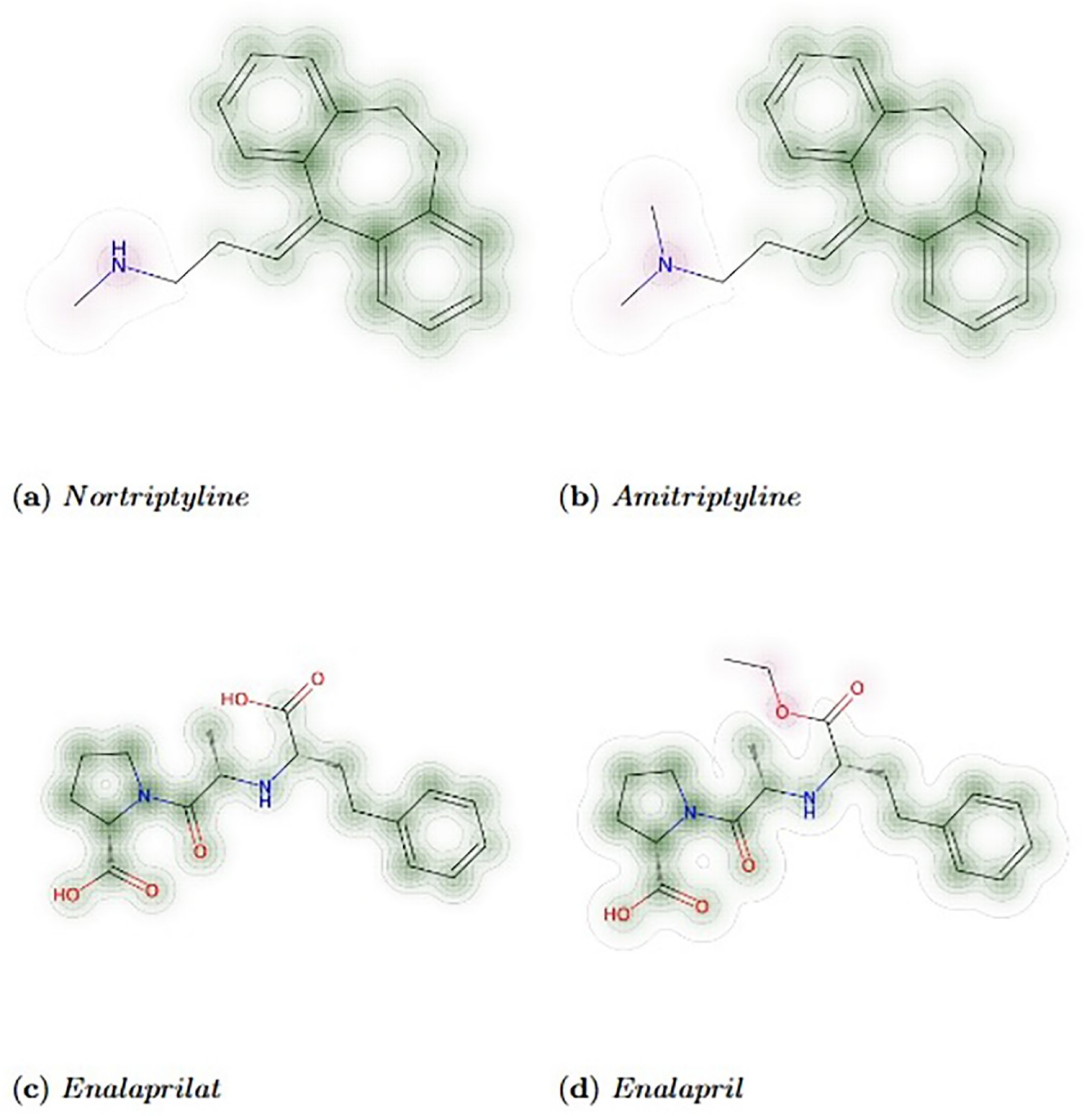


The preparatory work shown in Table 8 and Fig 13 illustrates two groups of drugs, both involving Drug A interacting with Drug B, and Drug C bearing molecular structural similarity to Drug A, for better understanding. Next, we will introduce this in three steps: First, we obtained the severity level of the interaction between Drug A and Drug B using the drug databases. Second, we identified Drug C, which can interact with Drug B, from the DDIs predicted by MDFLDRR and then calculated the molecular structural similarity between Drug C and Drug A. Third, based on the severity level of the interaction obtained in the first step and the molecular structure similarity obtained in the second step, we speculate on the severity level of the interaction between Drug C and Drug B. Here, the key point is that in most cases, there is more than one Drug C that can interact with Drug B and has high molecular structure similarity with Drug A identified in the second step. Therefore, extensive training is needed to output a sufficiently accurate severity level in the third step.

According to our calculations, the severity level of the interaction between Drug B and Drug C in the first group of drugs is MODERATE, which is consistent with the findings from the drug databases [46–50]. The severity level of the interaction between Drug B and Drug C in the second group of drugs is MINOR.

**Table 8:** Preparatory work for inferring the severity of drug interactions based on molecular structural similarity calculation.

Drug A	Drug B	Drug C	A & B Severity Level	C & A Similarity
Nortriptyline	Irbesartan	Amitriptyline	MO^2^‖MA^1^	0.9762
Enalaprilat	Venlafaxine	Enalapril	MI^1^	0.9084

^1^ represents evidence sourced from DrugBank Online,

^2^ represents evidence sourced from Drugs.com.

However, as there are no records(*N*.*A*.) in the drug databases, this result is for reference only.

By comparing and validating the severity levels obtained through our method with those from DrugBank Online and Drugs.com, we found that the correspondence provided in Table 4 matches exceptionally well with the severity levels given by the drug databases in Tables 5–7. This directly validates the feasibility of inferring the severity of drug interactions based on the similarity of drug molecular structures. Simultaneously, we also applied this method to unobserved DDIs predicted by MDFLDRR and marked their severity levels for reference.

The reason why different databases have different judgments on the severity of interactions between the same pair of drugs occurs because the assessment of the severity of drug interactions usually depends on clinical research and reports, including clinical trials of drugs, medical literature, patient reports and so forth [51]. However, there may sometimes be differences in these data sources, as research methods, patient samples, data interpretation and evaluation standards may vary [52].

### Complexity analysis

#### Experimental setup

Our MDFLDRR method was developed using Python. To ensure the stability of the experiments and facilitate comparison, we conducted all experiments on a computer equipped with a 64-bit Windows operating system. This computer has 32GB of random access memory and an Intel(R) Core(TM) i9–10980HK CPU @ 2.40GHz 3.10 GHz processor. Throughout the experiments, we kept the processor running at its maximum clock frequency to ensure that the experiments could be completed with the highest possible efficiency.

#### MDFLDRR time complexity

Given the context, we need to consider three things. First, we use *j*, which represents the number of different drugs considered. Second, we use *t*, which signifies the number of different drug features considered. Third, we use *y*, which denotes the highest dimension among the drug features considered. The time complexity required for one iteration is *O*(*j*^2^*t*^2^*y*). In fact, all of our experiments have converged within 9 iterations, thus MDFLDRR time complexity is *O*(*j*^2^*t*^2^*y*).

## Conclusions

In this research, we propose Multi-Drug Features Learning with Drug Relation Regularization (MDFLDRR), a method that learns from known DDIs and multiple drug features for predicting unobserved DDIs, especially those between cardiovascular drugs and antidepressants. By conducting comparative experiments with several of the most advanced methods currently, relevant results show that the performance of MDFLDRR significantly surpasses that of other comparison methods and can predict reasonable and authentic interactions for drugs that do not have any known interaction records. In addition, we specifically focus on predicting unobserved DDIs between cardiovascular drugs and antidepressants. For instance, DDIs between *Irbesartan* and other antidepressants, DDIs between *Venlafaxine* and other cardiovascular drugs, as well as DDIs between multiple cardiovascular drugs and multiple antidepressants. Subsequently, we validated the predicted DDIs by MDFLDRR through DrugBank Online and Drugs.com, obtaining a substantial amount of valid publicly available evidence support. Lastly, we not only discussed the time complexity of MDFLDRR but also addressed the issue of ascertaining the severity level of DDIs by generating molecular fingerprints using SMILES and calculating drug molecular structure similarity.

Regarding the existing shortcomings of MDFLDRR, we believe they are mainly reflected in the following two aspects. First, MDFLDRR has several tunable parameters, and a considerable amount of effort and time is required in the experiment to determine the values of these parameters for optimal results. Second, in the process of building the drug interaction prediction model, the number of drug features used by MDFLDRR is limited. Future research can consider adding more drug features to achieve better prediction accuracy and discover more DDIs that have not been observed in previous studies.

## Supporting information

S1 File (PDF)
