# Supplementary material for: Drug-Drug interactions prediction calculations between cardiovascular drugs and antidepressants for discovering the potential co-medication risks
Source: PLoS One. 2025 Jan 13;20(1):e0316021. doi: 10.1371/journal.pone.0316021 (PMC11730380; doi:10.1371/journal.pone.0316021)
Supplement: S1 File — (PDF) [file pone.0316021.s001.pdf]

Supplementary Materials

Core Pseudocode

Algorithm 1: MDFLDRR

Input: Drug Feature Matrices List Hs, Similarity Matrices List Ls, Drug-Drug Interaction Matrix J, Optimization Parameters θ, σ and λ, Optimization Precision $epsilon$, Maximum Iteration Count $max\_iteration$.

Output: Matrices List Zs, Matrix K, List $different\_Z$, Iteration Count t, Matrix P.

Initialize Zs /* A list used to store random matrices for each matrix in the input matrices list Hs */, $elds$ /* A list that provides regularization terms needed for updating $As[i]$ */.
for *i in Hs* **do**
    Add the random matrix of the i-th drug feature matrix to Zs;
    Create a row vector with all elements being 1 and add it to $elds$.
end
Initialize L /* A zero matrix of the same order as square matrix J */.
for *i in range (0, len(Hs) - 3)* **do**
    $L = L + \sigma \times Ls[i]$.
end
Initialize Zs_old, As, As_pos, As_neg, Bs, Bs_pos, Bs_neg /* Seven empty lists of the same length as Hs */, K, $different\_Z$, P.
for *t in range (0, max_iteration)* **do**
    Initialize $Y = J$;
    for *i in range (0, len(Hs))* **do**
        $Zs\_old[i] = Zs[i]$;
        $Y = Y + \theta \times (Hs[i])^T \times Zs[i]$.
    end
    Calculate P that satisfies the following equation, where E_J represents the identity matrix of the same order as matrix J:
    $L + (1 + \theta \times (len(Hs) - 1)) \times E_J \times P == E_J$; $K = P \times Y$;
    for *i in range (0, len(Hs))* **do**
        $As[i] = Hs[i] \times ((\theta \times E_J - (\theta^2 \times P^T)) \times (Hs[i])^T) + \lambda \times (elds[i])^T \times elds[i]$;
        $Bs[i] = \theta \times Hs[i] \times P \times J$;
        Replace NaN values in $As[i]$ and $Bs[i]$ with 0.0;
        $As\_pos[i] = (As[i] + |As[i]|)/2$, $As\_neg[i] = (|As[i]| - As[i])/2$;
        for *j in range (0, len(Hs))* **do**
            if *i != j* **then**
                $Bs[i] = Bs[i] + \theta^2 \times Hs[i] \times P^T \times (Hs[j])^T \times Zs[j]$.
            end
        end
        $Bs\_pos[i] = (Bs[i] + |Bs[i]|)/2$, $Bs\_neg[i] = (|Bs[i]| - Bs[i])/2$.
    end
    for *i in range (0, len(Hs))* **do**
        Update $Zs[i]$.
    end
    for *i in range (0, len(Hs))* **do**
        $different\_Z[i] = \|Zs[i] - Zs\_old[i]\| / \|Zs\_old[i]\|$.
    end
    if *mean (different_Z) ¡= epsilon* **then**
        return $[Zs, K, different\_Z, t]$.
    end
end
return $[Zs, K, different\_Z, P]$.

Algorithm 2: Drug Relation Regularization (DRR)

Input: Feature Matrix FM, Neighbor Number NN.
Output: Drug Proximity Relationship Matrix M.

Initialize $max\_iteration = 30$, $\theta = 3$, $X = FM$, $row\_num = X.shape[0]$,
$distance\_matrix = np.zeros((len(X), len(X)))$.
for *i in range (len(X))* **do**
  for *j in range (i+1, len(X))* **do**
    $distance\_matrix[i, j] = np.sqrt(np.sum(np.square(X[i, :] - X[j, :])))$.
  end
end
$distance\_matrix = distance\_matrix + distance\_matrix^T$.
$e = np.ones((row\_num, 1))$.
$distance\_matrix = distance\_matrix + np.diag(np.diag(e \times e^T \times float('inf')))$.
$nearst\_neighbor\_matrix = np.zeros((row\_num, row\_num))$.
for *i in range (row_num)* **do**
  $b = sorted(enumerate(distance\_matrix[i]), key = lambdax : x[1])$;
  $index = b[: (NN - 1)]$;
  for d *in* $index$ **do**
    $nearst\_neighbor\_matrix[i, d[0]] = 1.0$.
  end
end
$M = np.multiply(C, M)$, $\lambda = \theta \times e$, $P = X \times X^T + \lambda \times e^T$.
for *i in range (max_iteration)* **do**
  $Q = (np.multiply(C, M)) \times P$, $M = np.multiply(np.multiply(C, M), P)/Q$;
  for *i in range (len(M))* **do**
    for *j in range (len(M))* **do**
      if *M[i, j] == np.nan* **then**
        $M[i, j] = 0$.
      end
    end
  end
end
return M.

Algorithm 3: Compute and Pack Normalized Similarity Matrices

Input: Pathway Matrix PM, Enzyme Matrix EM, Target Matrix TM, Structure Matrix SM, Parameter p.
Output: Feature Similarity Matrices List All_{simi}.

Initialize $Path_{simi}$, $Enzy_{simi}$, Tar_{simi}, $Structure_{simi}$.
/ Continuing with Algorithm 2 /.
$Path_{simi} = DRR(PM, p)$, $Enzy_{simi} = DRR(EM, p)$.
$Tar_{simi} = DRR(TM, p)$, $Structure_{simi} = DRR(SM, p)$.
/ Normalize the matrices /.
$Path_{simi} = matrix\_normalize(Path_{simi})$.
$Enzy_{simi} = matrix\_normalize(Enzy_{simi})$.
$Tar_{simi} = matrix\_normalize(Tar_{simi})$.
$Structure_{simi} = matrix\_normalize(Structure_{simi})$.
Initialize $All_{simi} = [\,]$.
$All_{simi} = [Path_{simi}, Enzy_{simi}, Tar_{simi}, Structure_{simi}]$.
return All_{simi}.

Algorithm 4: Drug-Drug Interaction Intensity Prediction

Input: Similarity Matrices List All_{simi}, Training Set Feature Matrices Xs, Test Set Feature Matrices X_{test}, Drug-Drug Interaction Matrix J, Optimization Parameters θ, σ and λ, Optimization Precision $epsilon$, Maximum Iteration Count $max\_iteration$.

Output: Predicted Drug-Drug Interaction Intensity Matrix $Pred_{d\text{-}dii}$.

```
Initialize Ls = [] */ A list for storing Laplacian matrices /*.
for i in All_simi do
    nor_i = matrix_normalize(i); */ Normalize each matrix /*.
    L = σ × (I − nor_i); */ Compute each Laplacian matrix /*.
    Add the Laplacian matrix L to Ls.
end
*/ Continuing with Algorithm 1 /*.
Results = MDFLDRR(Xs, Ls, J, θ, σ, λ, epsilon, max_iteration).
Initialize Zs = [], Pred_d-dii */ A zero-filled matrix /*.
Zs = Results[0]. */ Extract Zs from Results /*.
for i in range (len(Xs)) do
    Pred_d-dii = Pred_d-dii + (X_test[i]^T × Zs[i]) / Σ(X_test[i]^T × Zs[i]).
end
return Pred_d-dii.
```
